# Oral administration of *Proteus mirabilis* damages dopaminergic neurons and motor functions in mice

**DOI:** 10.1038/s41598-018-19646-x

**Published:** 2018-01-19

**Authors:** Jin Gyu Choi, Namkwon Kim, In Gyoung Ju, Hyeyoon Eo, Su-Min Lim, Se-Eun Jang, Dong-Hyun Kim, Myung Sook Oh

**Affiliations:** 1 Department of Life and Nanopharmaceutical Sciences, Graduate School, Kyung Hee University, 26, Kyungheedae-ro, Dongdaemun-gu, Seoul, 02447 Republic of Korea; 2 Department of Oriental Pharmaceutical Science, College of Pharmacy and Kyung Hee East-West Pharmaceutical Research Institute, Kyung Hee University, 26, Kyungheedae-ro, Dongdaemun-gu, Seoul, 02447 Republic of Korea

## Abstract

Recently, studies on the relationship between gut dysbiosis and Parkinson’s disease (PD) have increased, but whether a specific gut bacterium may cause PD remains unexplored. Here, we report, for the first time, that a specific gut bacterium directly induces PD symptoms and dopaminergic neuronal damage in the mouse brain. We found that the number of *Enterobacteriaceae*, particularly *Proteus mirabilis*, markedly and commonly increased in PD mouse models. Administration of *P. mirabilis* isolated from PD mice significantly induced motor deficits, selectively caused dopaminergic neuronal damage and inflammation in substantia nigra and striatum, and stimulated α-synuclein aggregation in the brain as well as in the colon. We found that lipopolysaccharides, a virulence factor of *P. mirabilis*, may be associated in these pathological changes via gut leakage and inflammatory actions. Our results suggest a role of *P. mirabilis* on PD pathogenesis in the brain.

## Introduction

Parkinson’s disease (PD) is a progressive neurological disease, accompanied by motor symptoms, such as rigidity, bradykinesia, tremor, and postural abnormalities, due to degeneration of dopaminergic and γ-aminobutyric acid neurons in the basal ganglia, including substantia nigra (SN)^1–3^. PD has key pathological features, including nigrostriatal Lewy bodies, composed primarily of accumulated α-synuclein and neuroinflammatory responses characterized by reactive gliosis^4^. Up to 60% of dopaminergic neurons have already been lost by the time PD is diagnosed based on motor impairment, but the current therapeutics, consisting mainly of dopamine replacement drugs such as levodopa and several dopamine agonists, are restricted to simply relieving the clinical symptoms. Indeed, these drugs don’t cure the disease and even cause severe adverse events like dyskinesia and dopamine dysregulation syndrome^5–7^. Thus, there is a continuing need for a novel approach in the development of diagnostics and therapeutics for PD^4,8^.

Before the onset of motor symptoms, PD patients suffer from many non-motor symptoms, including olfactory, gastrointestinal, cardiovascular, and urological problems, indicating that PD may start from non-neurocentric tissues outside the brain^9,10^. Previous studies on pathological changes in the intestine of PD patients and PD animal models suggest the possibility that gut alteration is associated with PD pathogenesis. Both accumulated α-synuclein and 3-nitrotyrosine, which induce apoptotic cell death in dopaminergic neurons^11^, are increased significantly in colonic submucosal nerve fibers of early PD subjects versus healthy controls^12,13^. Accumulated α-synuclein was observed in the intestine and brain of Thy1-α-syn or A53T mutant α-syn transgenic mice before the onset of motor signs^14,15^. Moreover, it was shown experimentally that monomeric, oligomeric, and fibrillar α-synuclein forms can migrate from the gut to the brain via the vagal nerve^16^. From a different perspective, studies on permeability and inflammation in the intestines of PD patients and PD animal models have been reported. Both intestinal penetrability and lipopolysaccharide (LPS)-binding protein in plasma were increased significantly in PD patients^17^. Colonic inflammation was upregulated by pro-inflammatory cytokine production and glial cell expression in PD patients^18^. Increased intestinal permeability and α-synuclein accumulation were observed in the large intestine of PD mice administered LPS systemically^19^. Levels of pro-inflammatory cytokines, such as tumor necrosis factor-α (TNF-α) and interleukin-1β (IL-1β), were also increased in colonic tissues from 6-hydroxydopamine (6-OHDA)-lesioned PD rats^20^. Taken together, these previous studies suggest that intestinal pathological changes may influence the pathogenesis of PD.

Gut microbial imbalance is an important factor in intestinal pathology^21^. In fact, gut microbial changes in PD patients have been published. In 16 S ribosomal RNA (rRNA) gene pyrosequencing analysis in the fecal microbiota of PD patients, a higher abundance of the *Enterobacteriaceae* family was seen in non-tremor dominant PD patients, who were observed to have faster progression, worse outcomes, and more severe colonic α-synuclein pathology^12,22^. Several experimental studies revealed that exposure to curli, a bacteria-producing functional amyloid protein, enhanced α-synuclein aggregation both in rats and in nematodes, and gut microbiota regulate α-synuclein dependent neuroinflammation and motor dysfunction in α-synuclein overexpressing PD mice^23,24^. Although these previous studies raise the possibility that intestinal pathology by alteration of gut microbiota may be involved in the onset or aggravation of PD, it still remains unexplored what gut bacterial strains can cause PD.

We therefore first explored the claim that treatment with a specific gut bacterium may induce motor deficits and nigrostriatal dopaminergic neuronal damage. To find a specific gut bacterium, we measured the number of bacterial colonies at the family level in the feces of PD animal models and identified this bacterium from the increased bacterial family. Firstly, we examined whether this bacterium exacerbates PD symptoms and dopaminergic neuron death at the premotor phase. Then, we observed motor behaviors and brain tissues, including SN, striatum (ST), hippocampus (HP), and cortex after the oral administration of this bacterium to normal mice. We also examined how this bacterium injured dopaminergic neurons by assessing the changes of inflammatory factors and α-synuclein in the brain as well as in the colon.

## Results

### A specific gut bacterium, *Proteus mirabilis*, is identified from the genera *Proteus* which is isolated from the commonly increased *Enterobacteriaceae* family in the feces of PD mice models

**Figure 1:**
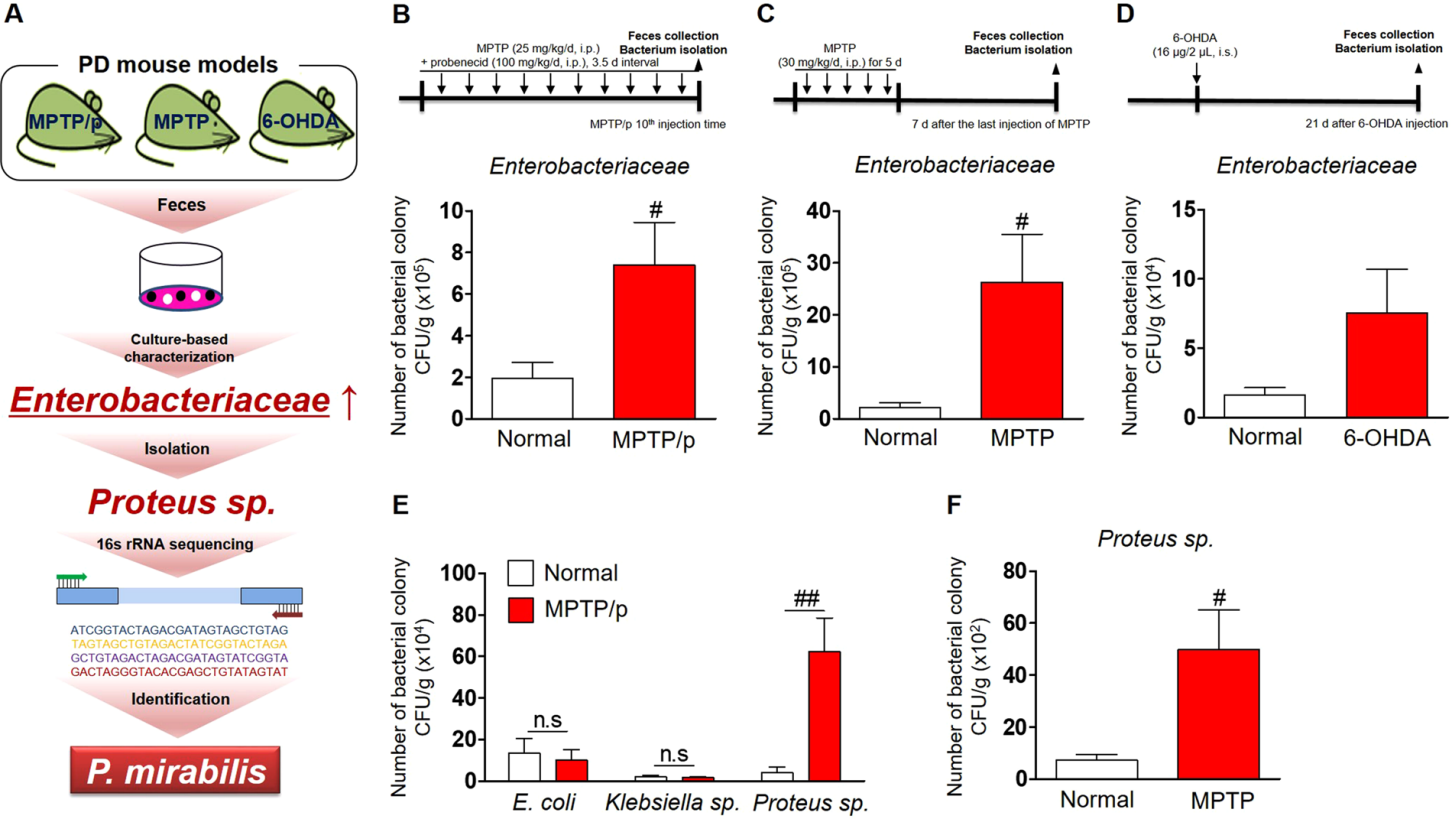
*P. mirabilis* is an isolated bacterium from the increased bacterial colonies in PD animal models. (**A**) Schematic diagram of isolation and identification processes of *P. mirabilis*. (**B**–**D**) The colonies of *Enterobacteriaceae* were increased in the feces of MPTP/p, MPTP, and 6-OHDA-induced PD mice compared with the normal group, respectively. (**E**) The colonies of *Proteus sp*. in MPTP/p mice were only increased compared with the normal group whereas those of *E. coli* and *Klebsiella sp*. were no different from each normal group. (**F**) The colonies of *Proteus* sp. in MPTP mice were also increased compared with the normal group. Values were expressed as means ± SEM. #p < 0.05 and ##p < 0.01 vs. each normal group (unpaired t-test; n = 4). n.s; not significant, CFU; colony-forming unit.

*Enterobacteriaceae* is a representative pathogenic bacterial family that changes in disease states^25^. It has been reported that the *Enterobacteriaceae* family triggers colonic inflammatory conditions and is distinctly increased in the colon of PD patients^22,26–28^. As shown in Fig. 1A, we explored whether these bacterial colonies changed in PD mice models induced by 1-methyl-4-phenyl-1,2,3,6-tetrahydropyridine (MPTP), MPTP/probenecid (MPTP/p), and 6-OHDA toxicity. The number of *Enterobacteriaceae* increased significantly compared with those of each control group for three PD mice models (Fig. 1B–D). We found that, in the *Enterobacteriaceae*, the number of *Proteus sp*. was increased significantly in MPTP/p mice versus the normal group, whereas *Escherichia coli* (*E. coli*) and *Klebsiella sp*. hardly differed from each normal group (Fig. 1E). We confirmed that *Proteus sp*. was increased significantly in MPTP-induced PD mice (Fig. 1F). Then, we identified the increased *Proteus sp*. as *Proteus mirabilis* by 16 S rRNA gene sequencing (Table S1). Next, we assessed whether treatment with *P. mirabilis* influenced the mouse brain as well as colon.

### Administration of *P. mirabilis* exacerbates striatal dopaminergic neuronal damage and induces motor deficits at the premotor phase of PD

**Figure 2:**
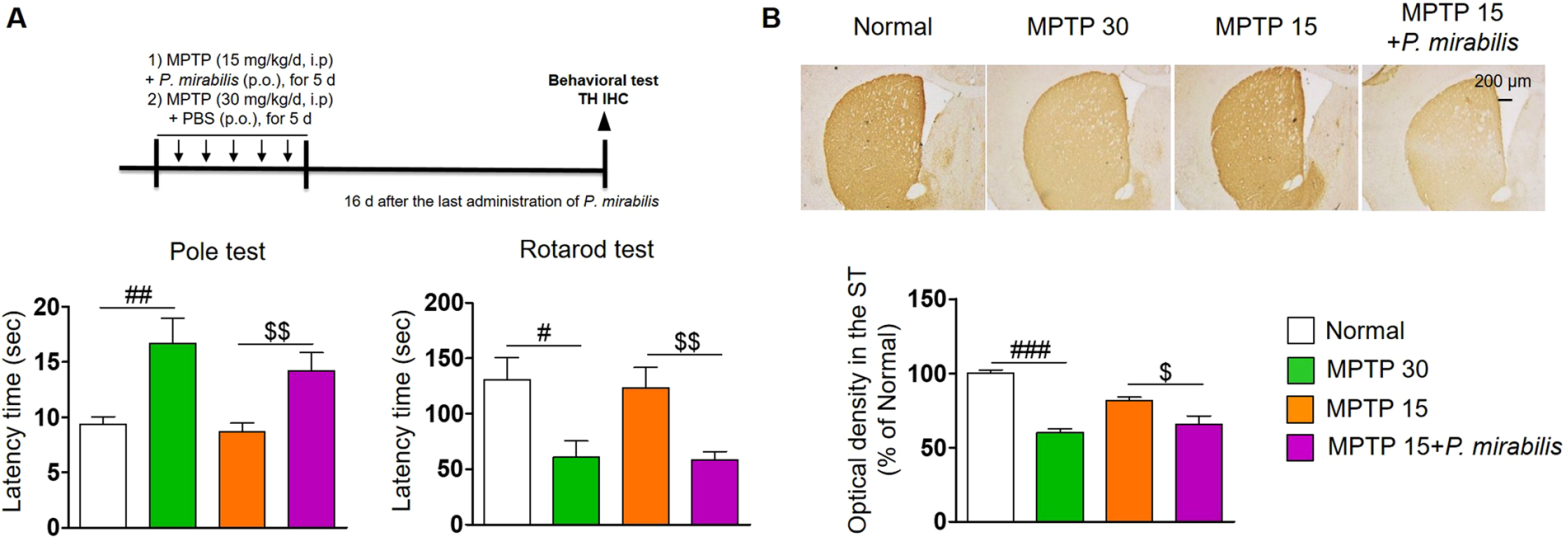
Aggravation of MPTP-induced motor impairment and dopaminergic neuronal damage by *P. mirabilis* treatment. (**A**) *P. mirabilis* exacerbated MPTP-induced motor deficits (pole test and rotarod test; n = 10). (**B**) *P. mirabilis* also reinforced MPTP-induced striatal dopaminergic neuronal damage compared with the premotor stage induced by MPTP (15 mg/kg/d, i.p., for 5 d) injection (MPTP 15; n = 5). Values were expressed as means ± SEM. #p < 0.05, ##p < 0.01, and ###p < 0.001 vs. normal group (unpaired t-test); $p < 0.05 and $$p < 0.01 vs. the MPTP 15 group (unpaired t-test). IHC; immunohistochemistry.

To examine whether *P. mirabilis* impairs motor function during the premotor stages of PD, we treated MPTP (15 mg/kg/day, *i.p*., for 5 days)-injected mice with *P. mirabilis*. The motor function of the mice injected with MPTP at 15 mg/kg alone was not impaired, while the mice co-treated with *P. mirabilis* showed significant movement impairments, similar to that of PD mice induced by MPTP (30 mg/kg/day, *i.p*., for 5 days) injection, which showed severe damage in motor function (Fig. 2A). Moreover, *P. mirabilis* treatment reinforced the MPTP-induced dopaminergic neuronal degeneration in the ST (Fig. 2B). These results show the possibility that *P. mirabilis* may be concerned with PD pathology.

### *P. mirabilis* treatment impairs motor function, not affecting memory function

**Figure 3:**
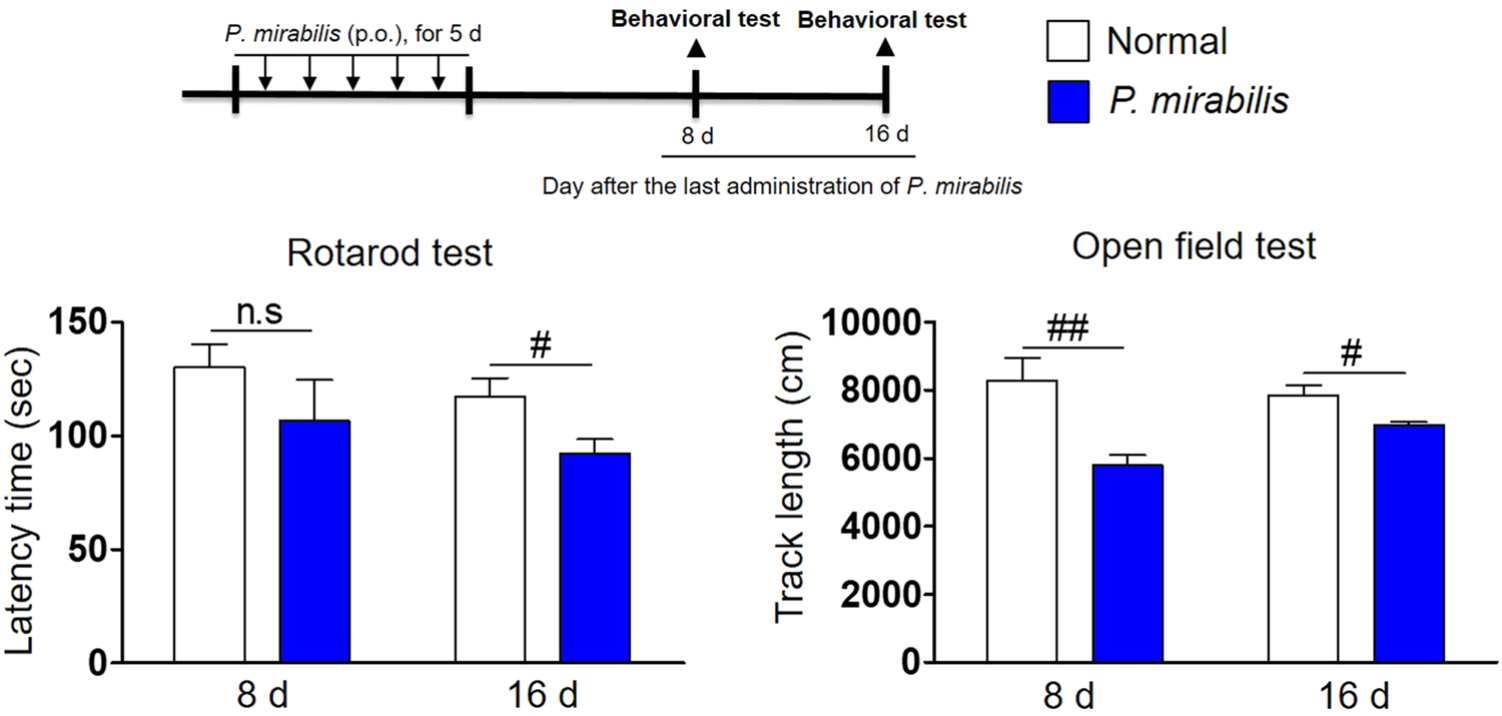
*P. mirabilis* treatment impairs motor function in mice. Two behavioral tests (rotarod test and open field test) were performed at 8^th^ and 16^th^ day after last administration of *P. mirabilis*. Values were expressed as means ± SEM. #p < 0.05 and ##p < 0.01 vs. each normal group (unpaired t-test; n = 8). n.s; not significant.

We explored whether treatment with *P. mirabilis* alone induces movement deficits in normal mice. We found that motor ability in the rotarod test was significantly impaired at the 16^th^ day after the last administration of *P. mirabilis* (Fig. 3). The locomotor activity in the open field test was also significantly decreased at the 8^th^ and 16^th^ days after last administration of *P. mirabilis* (Fig. 3). On the other hand, *P. mirabilis* administration did not affect memory function (Fig. S1). These results show that *P. mirabilis* may induce motor impairment while it has no impact on the memory function.

### *P. mirabilis* treatment selectively damages dopaminergic neurons in the brain

**Figure 4:**
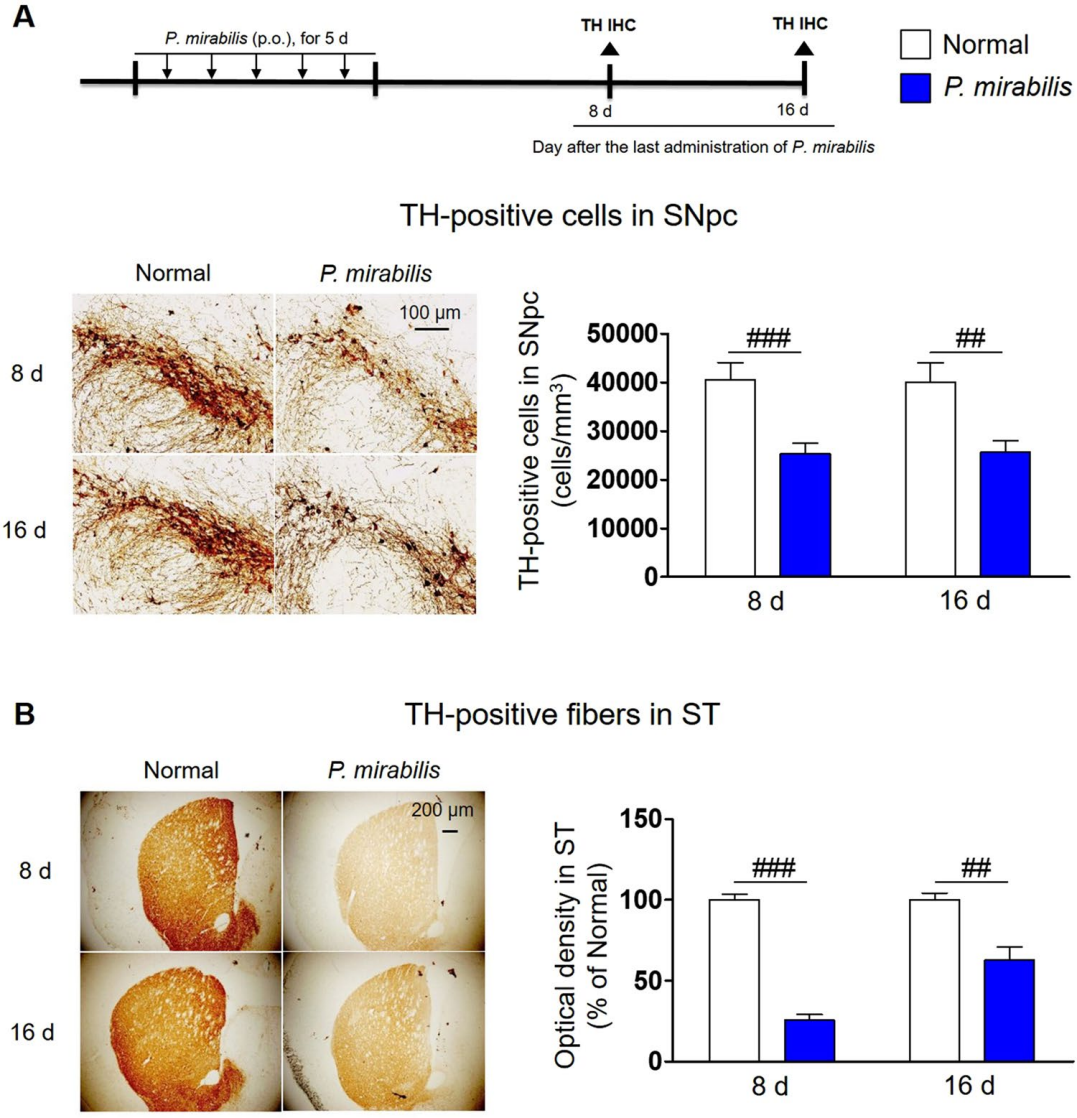
Dopaminergic neuronal damage in normal mice with PM administration. (**A**) Dopaminergic neuronal cell death was shown in the SNpc of mice brain at 8^th^ and 16^th^ day after PM treatment. (**B**) The loss of dopaminergic terminals was also shown in the ST of mice brain at the same time points. Values were expressed as means ± SEM. ##p < 0.01 and ###p < 0.001 vs. each normal group (ANOVA following Tukey’s post hoc test; n = 8). IHC; immunohistochemistry.

To examine whether dopaminergic neuronal damage in the brain was provoked by *P. mirabilis* itself, we analyzed histological changes at the 8^th^ and 16^th^ days after *P. mirabilis* administration for 5 days. Surprisingly, severe damage in dopaminergic neurons was observed in both the SN (Fig. 4A) and the ST (Fig. 4B) in the *P. mirabilis*-treated mice versus the normal group, while hippocampal and cortical neurons were not damaged by *P. mirabilis* treatment (Fig. S2). These results suggest that *P. mirabilis* causes damages to brain dopaminergic neurons selectively.

### *P. mirabilis* treatment induces neuroinflammation in the nigrostriatal brain regions

**Figure 5:**
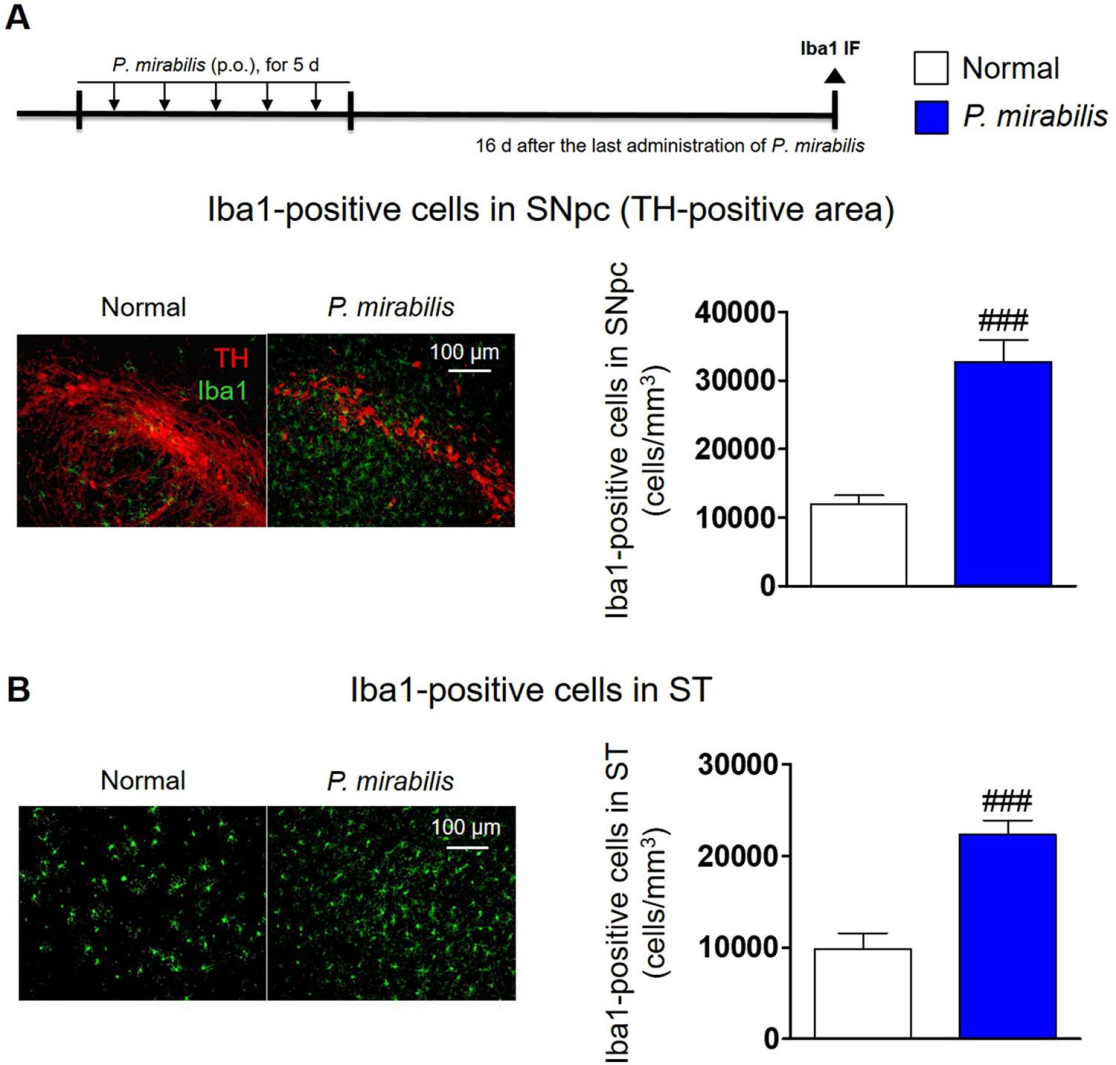
Glial activation in normal mice with *P. mirabilis* administration. (**A**) The number of activated microglia (Iba1 (green)-positive cells) was significantly increased in the SNpc of the brain (TH (red)-positive area) after *P. mirabilis* treatment, respectively. (**B**) This tendency was also consistent with that found in the ST region. Values were expressed as means ± SEM. ###p < 0.001 vs. normal group (ANOVA following Tukey’s post hoc test; n = 8). IF; immunofluorescence.

To demonstrate how *P. mirabilis* treatment selectively induces dopaminergic neuronal damage in the brain, we examined whether *P. mirabilis* provokes neuroinflammation. Interestingly, the number of activated microglia was significantly increased in the SN (Fig. 5A) and ST (Fig. 5B) of *P. mirabilis*-treated mice brains at the 16^th^ day after *P. mirabilis* treatment compared with the normal group, but this phenomenon was not observed in the hippocampal and cortical regions of the brain (Fig. S3). Thus, this result indicates that *P. mirabilis*-induced brain damage is specific and selective, particularly in dopaminergic neurons-enriched brain areas.

### LPS elevated by *P. mirabilis* treatment induces colonic barrier disruption and inflammation

**Figure 6:**
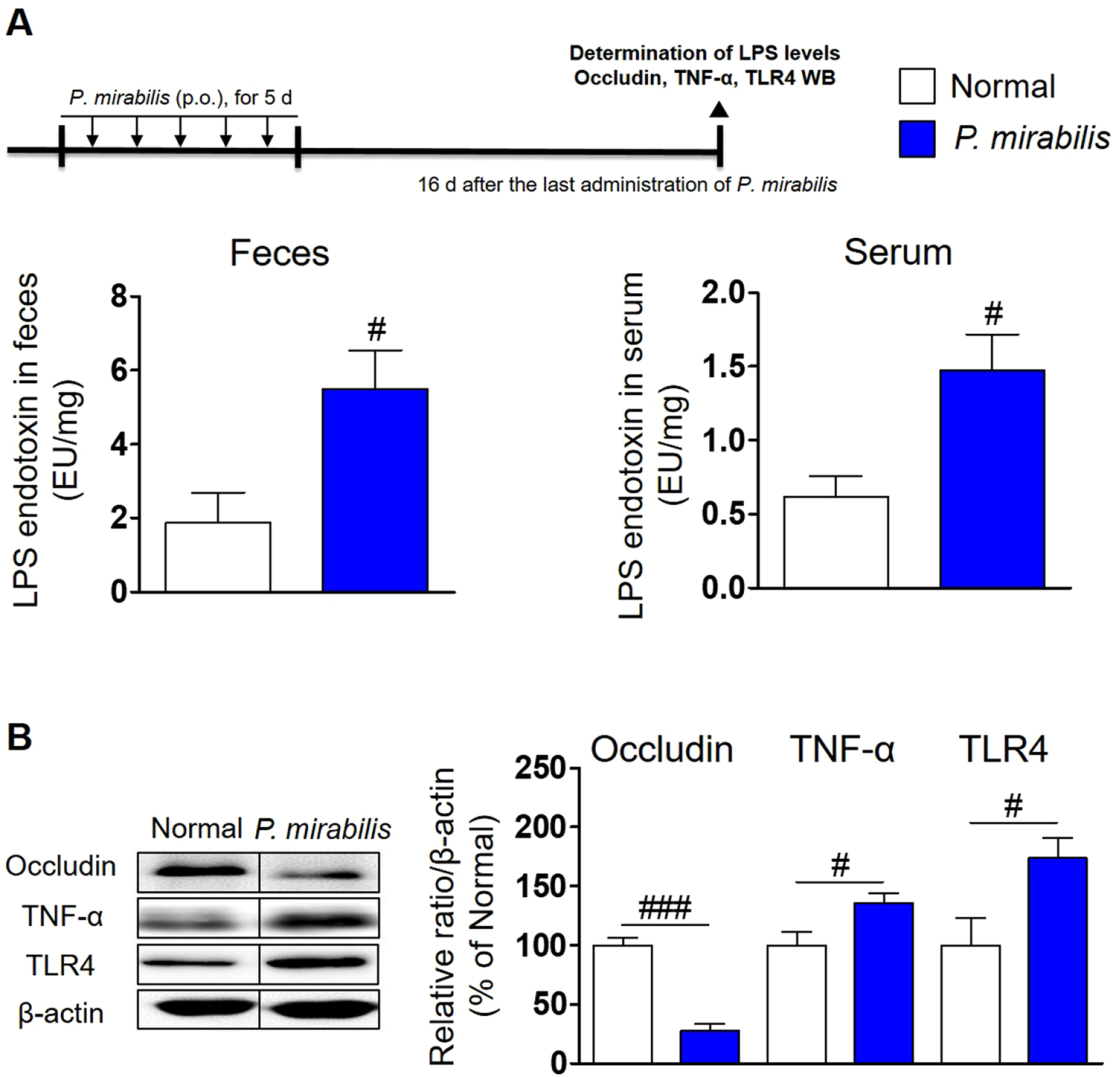
*P. mirabilis* treatment stimulates the elevation of fecal and serum LPS levels, colonic barrier disruption, and inflammation. (**A**) The levels of endotoxin LPS were elevated in the feces and serum of mice after *P. mirabilis* administration, respectively. The blots were processed in parallel using the samples derive from the same experiment. (**B**) *P. mirabilis* treatment induces the decrease of the epithelial barrier tight junction protein (occludin) and release of inflammatory cytokines (TNF-α) via activation of TLR4 by LPS in distal colon. Values were expressed as means ± SEM. #p < 0.05 and ###p < 0.001 vs. each normal group (unpaired t-test; n = 4). WB; western blotting.

We were curious as to how *P. mirabilis* treatment induced dopaminergic neurodegeneration and inflammation in the brain. Because *P. mirabilis* produces LPS^29^, which can induce inflammatory conditions through producing various proinflammatory mediators^30^, we considered that LPS may induce inflammation in the gut and brain, so we measured LPS levels in feces and serum after *P. mirabilis* treatment. LPS levels in feces and serum were significantly increased at the 16^th^ day after the treatment with PM, respectively (Fig. 6A). The increased colonic translocation of LPS derived from *Enterobacteriaceae* shows epithelial barrier disruption and stimulation of intestinal immune response to produce cytokines, which could move into the brain and give rise to brain inflammation and apoptotic neuronal cell death^31–34^. We explored whether *P. mirabilis* treatment induced pathological changes in the colon. We found that *P. mirabilis* treatment significantly reduced the protein level of occludin, a tight junction protein of the epithelial barrier in the colon, compared with the normal group at the 16^th^ day after *P. mirabilis* administration (Fig. 6B). We found the increased release of the inflammatory cytokine TNF-α in colon of *P. mirabilis*-treated mice (Fig. 6B). Mice treated with *P. mirabilis* also exhibited the significant overexpression of toll-like receptor 4 (TLR4), a LPS binding receptor, in the colon (Fig. 6B). These results show that *P. mirabilis*-derived LPS causes pathological conditions in the colon, including epithelial barrier disruption and inflammation.

### Intra-rectal injection of LPS derived from *P. mirabilis* induces inflammation in the nigrostriatal regions of the brain

**Figure 7:**
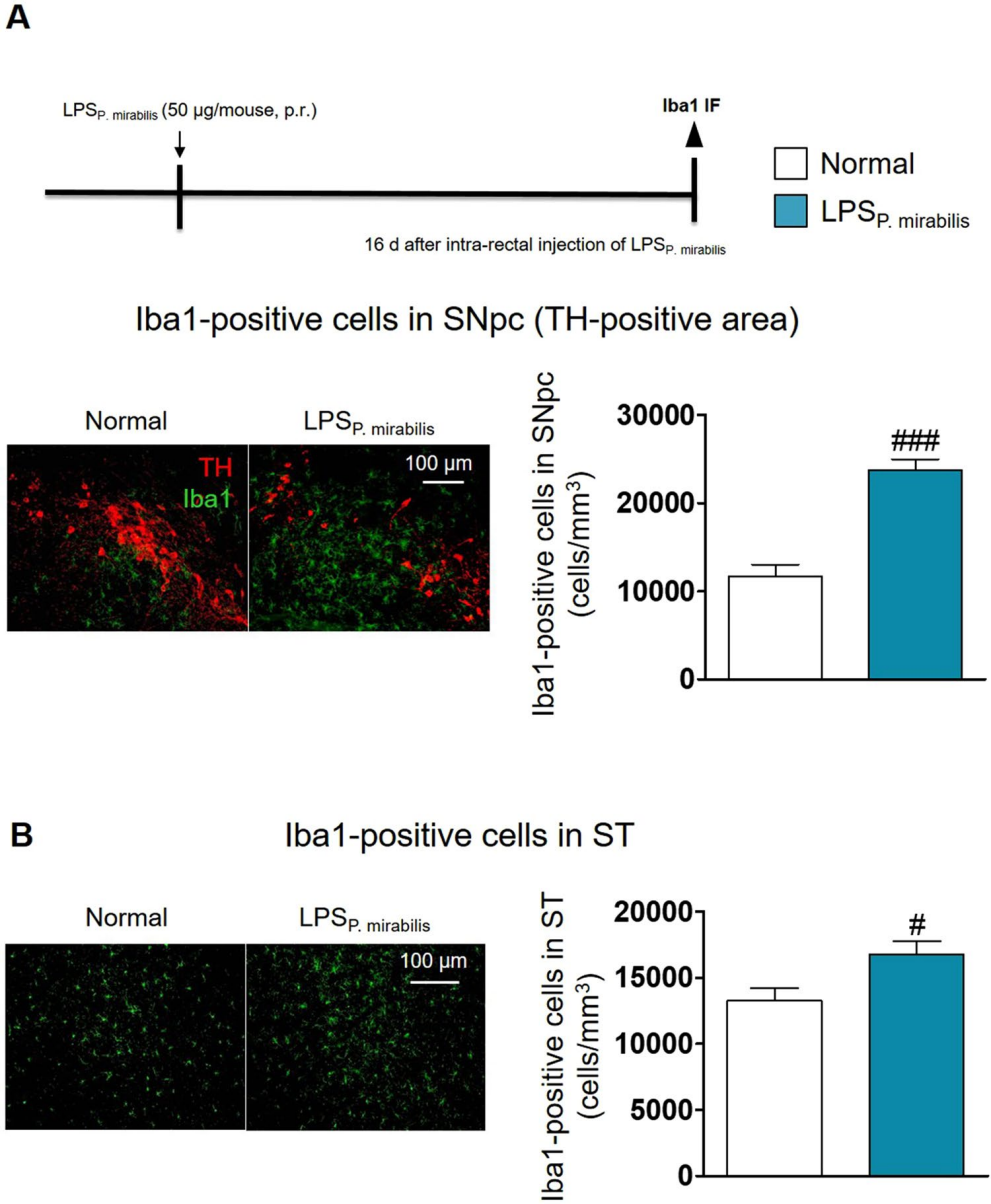
LPS_P. mirabilis_ induces inflammation in the nigrostriatal regions of the brain. (**A**, **B**) Microglial activation (Iba1 (green)-positive cells) was increased in the SNpc (TH (red)-positive area) and ST of mice with intra-rectal administration of LPS_P. mirabilis_, respectively. Values were expressed as means ± SEM. #p < 0.05 and ###p < 0.001 vs. each normal group (unpaired t-test; n = 4). IF; immunofluorescence.

We investigated whether LPS purified from *P. mirabilis* (LPS_P. mirabilis_) is directly involved in the neuroinflammation in mice with intra-rectal injections of LPS_P. mirabilis_. Microglial activation was significantly increased in the nigrostriatal regions such as the SN (Fig. 7A) and ST (Fig. 7B), while it exhibited no differences in the hippocampal and cortical regions at the 16^th^ day after treatment with LPS_P. mirabilis_ (Fig. S4). This result supports that LPS may be involved in the *P. mirabilis*-induced selective brain damage in the dopaminergic neurons.

### *P. mirabilis* treatment triggers the aggregation of α-synuclein in the colon as well as in the brain

**Figure 8:**
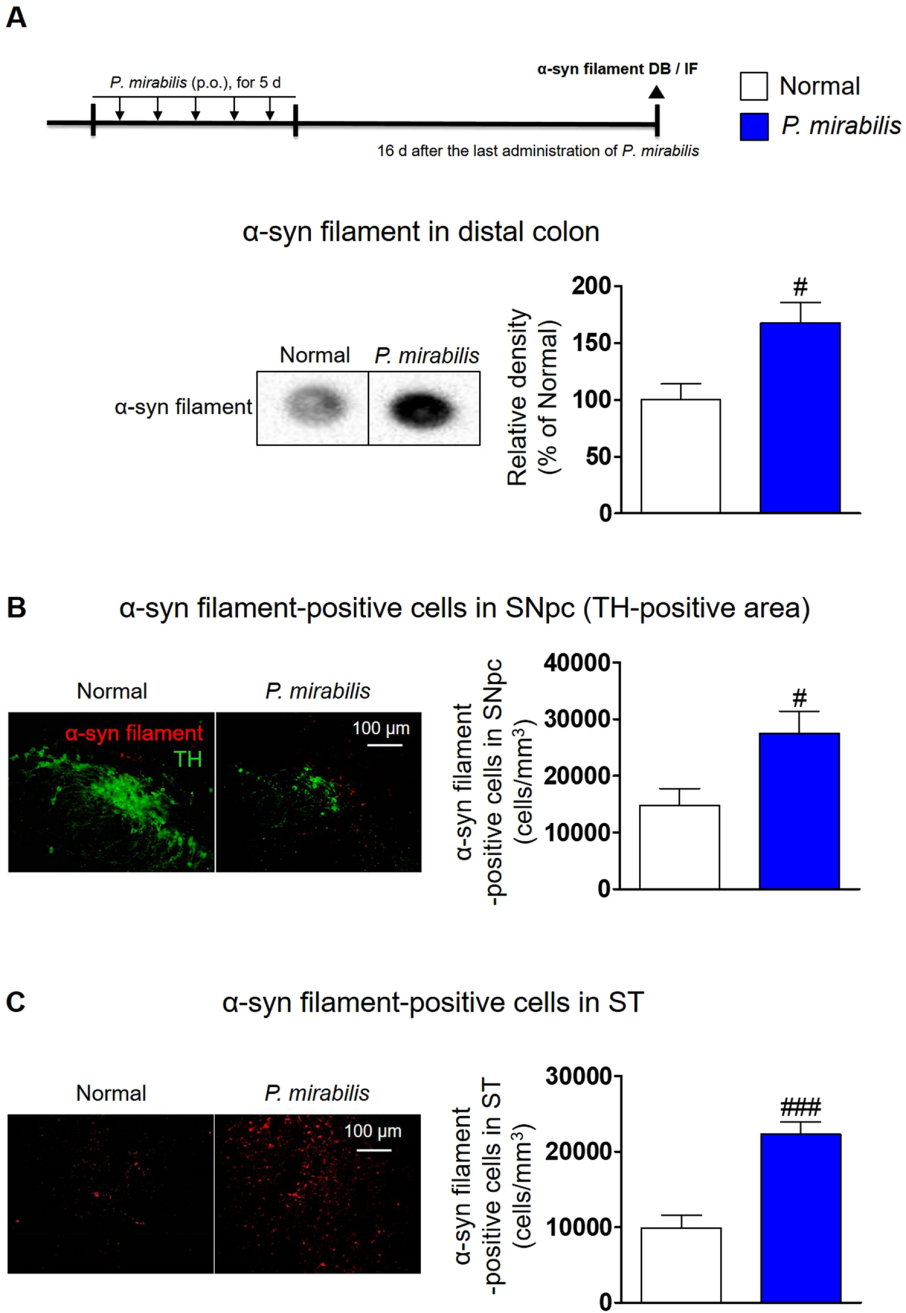
*P. mirabilis* treatment triggers aggregation of α-synuclein both in the colon and in the brain. The blots were processed in parallel using the samples derive from the same experiment. (**A**) The protein levels of α-synuclein filament was overexpressed in the distal colon of *P. mirabilis* -treated mice (n = 3). (**B**,**C**) *P. mirabilis* treatment stimulated immunoreactivity of α-synuclein filament (red) in the SNpc (TH (green)-positive area) and ST brain regions, respectively. Values were expressed as means ± SEM. #p < 0.05 and ###p < 0.001 vs. each normal group (unpaired t-test; n = 4). DB; dot blotting, IF; immunofluorescence, α-syn; α-synuclein.

It has been reported that the upregulation of α-synuclein expression occurs concurrently in enteric neurons as well as in dopaminergic neurons in PD^35,36^. Moreover, it has been shown that α-synuclein in enteric neurons moves into the SN via the vagus nerves in animal models^16,37^. Thus, we considered the view that *P. mirabilis* may induce α-synuclein aggregation in the colon and that such aggregated α-synuclein may move into the brain and cause neuroinflammation and dopaminergic neuronal cell death. To assess whether *P. mirabilis* treatment stimulates α-synuclein expression in neuronal cells, we assessed mRNA levels of α-synuclein after *P. mirabilis* treatment in SH-SY5Y cells. We found that the mRNA levels of α-synuclein were increased significantly in the *P. mirabilis*-treated group in a manner similar to those of the LPS-treated group when compared with untreated cells (Fig. S5). This change indicates that *P. mirabilis* may modulate the expression of α-synuclein. In this regard, we examined whether *P. mirabilis* treatment stimulates the overexpression of α-synuclein monomer in the colon and brain, but we concluded that it did not show a significant difference compared with normal group (Fig. S6). We focused on the change of α-synuclein aggregates that are toxicant forms of α-synuclein in the colon and brain. We investigated whether *P. mirabilis* treatment may stimulate the aggregation of α-synuclein in the colon. We found a significant increase of α-synuclein filaments (an aggregated form) in the distal colon at the 16^th^ day after *P. mirabilis* administration compared with the normal group (Fig. 8A). The immunoreactivity of α-synuclein filaments was significantly stronger than that of the normal group in the SN (Fig. 8B) and ST (Fig. 8C), respectively. These results suggest that *P. mirabilis* activates notable aggregation of α-synuclein both in the colon and in the brain, particularly in the brain regions of the nigrostriatal pathway. We also suggested the potential that aggregated α-synuclein may migrate from colon to brain via vagus nerve, showing that *P. mirabilis* treatment did not induce the aggregation of α-synuclein in SN of vagotomized (VGX) mice while *P. mirabilis* only treatment increases the levels of aggregated α-synuclein (Fig. S7).

## Discussion

**Figure 9:**
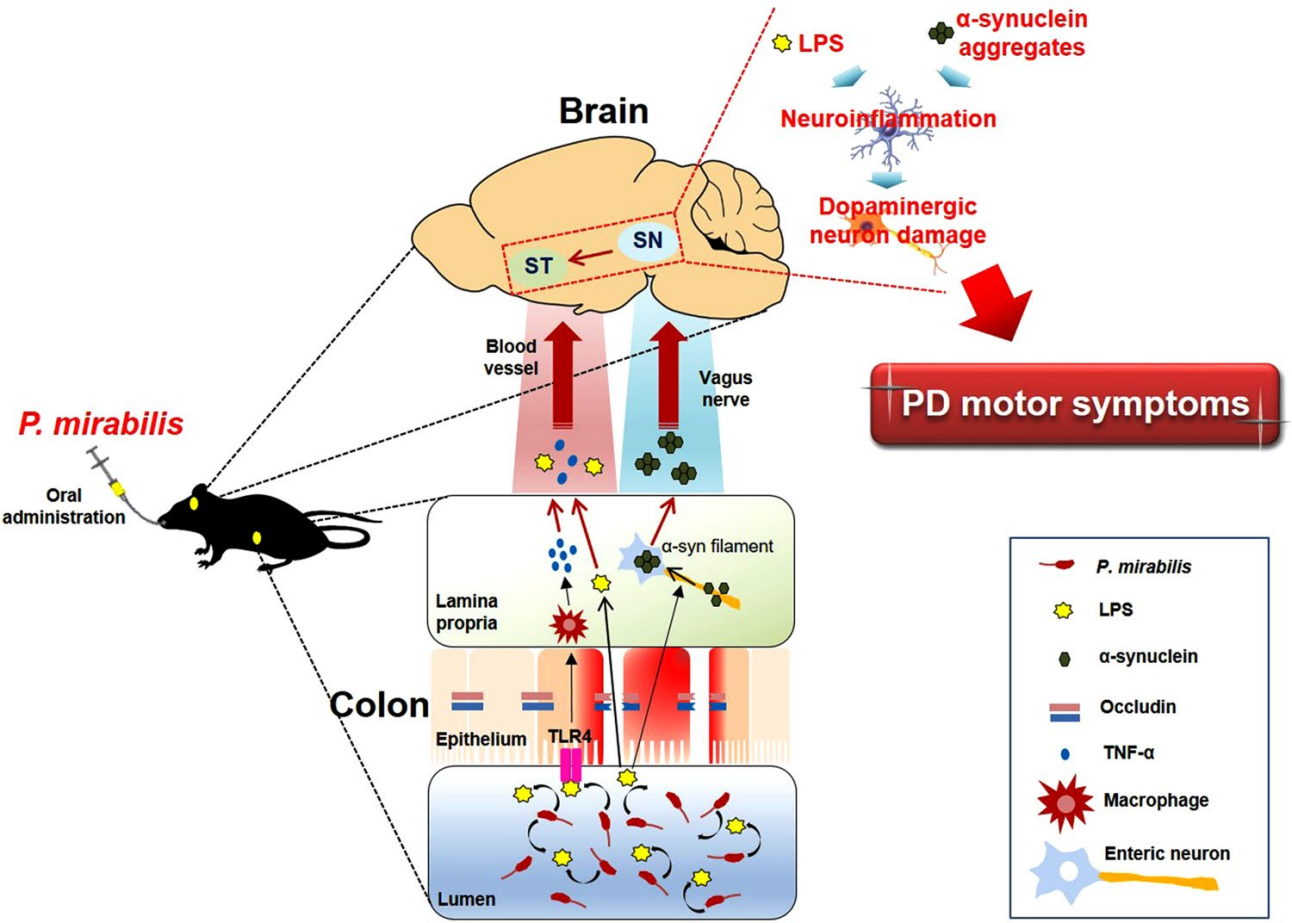
Schematic summary of *P. mirabilis*-induced PD pathogenesis in the colon and in the brain.

There is increasing evidence that the intestinal microbiota may regulate behavior and neuronal function, affecting neurological disorders or possibly attenuating them. Goehler *et al*. reported that *Campylobacter jejuni*, a common food-poisoning bacterium, induces anxiety-like behavior and c-fos protein expression in the amygdala and nucleus of the solitary tract, which process visceral and autonomic information^38^. Hsiao *et al*. showed that 4-ethylphenylsulfate, the most markedly increased metabolite from the gut microbiota in maternal immune activation-induced autism mice, induced abnormal autism-like behaviors, whereas commensal *Bacteroides fragilis* treatment attenuated this behavior^39^. In the case of PD, a recent evidence reported that short chain fatty acids produced by gut microbiota promote α-synuclein-mediated gliosis and PD motor symptoms^23^. As shown in Fig. 9, we demonstrated that a specific gut bacterium, *P. mirabilis*, was involved in the pathogenesis in a mouse model of PD.

First, we observed that gut microbiota dysbiosis had occurred regardless of toxin type in the colons of neurotoxin-induced PD mice. The bacterial colonies of *Enterobacteriaceae* family were significantly higher than those of each corresponding normal group. Then, we determined the genus of increased bacterial colonies as *Proteus* and it was identified as *P. mirabilis*. Interestingly, this result is consistent with a recent report that *Proteus* was a remarkably increased genus of the bacteria in α-synuclein overexpressing mice with intestinal dysbiosis induced by fecal transplantation from PD patients^23^.

Thus, our result is consistent with the previous study that *Enterobacteriaceae* increased significantly in PD patients^22^. Although *E. coli* is the most representative bacterium in the *Enterobacteriaceae* family and its endotoxin increases intestinal permeability and α-synuclein levels^17^, it showed no change in PD mice, whereas *P. mirabilis* showed levels increased over 10-fold compared with the normal group in our study. Before now, there has been no clinical report on the quantification of *E. coli* and *P. mirabilis* in the intestine, but recently it was reported that *P. mirabilis* was significantly higher in the urine of PD patients than in controls^40,41^. In that study, it was suggested that *P. mirabilis* was a causative bacterium of ‘purple urine bag syndrome’, increasing urinary indoxyl sulfate, a bacteria-generated metabolite. It was also shown that *P. mirabilis* acts as an inducible bacterium in pathological colonic changes. *P. mirabilis* stimulates robust IL-1β production in response to dextran sulfate sodium-induced colitis, mediated by the NOD-like receptor family pyrin domain-containing 3 inflammasome activation^42^. It could also trigger pathological colonic changes, characterized by colonic barrier disruption and elevated TNF-α levels as a key feature in mice with T-bet^−/−^RAG2^−/−^ ulcerative colitis^26^. Additionally, *P. mirabilis* was demonstrated to be one of the causative bacteria in brain infection, according to some case reports^43–45^. These previous studies suggest that *P. mirabilis* may induce pathological status in both the colon and the brain. Thus, we hypothesized that *P. mirabilis* may be pathogenic to dopaminergic neurons, and then we evaluated whether *P. mirabilis* treatment could exacerbate or induce PD-like pathological characteristics in mice. This bacterium worsened the motor symptoms and dopaminergic neuronal damage in mice at the premotor phase of PD induced by MPTP toxicity. Moreover, the treatment of this bacterium alone impaired motor function, induced severe dopaminergic neuronal damage, and activated glial cells in the SN and ST of normal mice. These results suggest that *P. mirabilis* may be involved in the pathogenesis of PD.

Next, we sought to examine how this bacterium affects brain damage. Because *P. mirabilis* is a gram-negative pathogenic bacterium that produces LPS endotoxin^29^, we considered that LPS derived from *P. mirabilis* in the colon could move to the periphery, and then induce neuroinflammation. We found that LPS levels in feces were elevated significantly at the 16^th^ day after *P. mirabilis* treatment, and the levels in serum increased consistently. These results suggested the possibility that *P. mirabilis*-generated LPS may be transferred from the colon to the brain via blood circulation, showing our results that microglial activation was observed in the SN and ST of mice performed intra-rectal injections of LPS_P. mirabilis_. This is consistent with the report of Qin *et al*. that increased inflammatory cytokines by systemic injection of LPS transferred inflammatory reactions from periphery to brain inducing microglial activation and dopaminergic neuronal damage in the SN^46^.

It has been reported previously that LPS derived from bacteria impairs the colonic barrier by reducing tight junction proteins such as occludin, and contributes to the release of TNF-α, which is stimulated by T helper 1 cell differentiation following TLR4-mediated macrophage activation in the lamina propria of the colon^47–49^. In this study, colonic barrier disruption and elevated TNF-α levels via TLR4 activation were seen after *P. mirabilis* administration. Clairembault and colleagues reported that expression of colonic epithelial barrier tight junction proteins was significantly lower in biopsy tissues from PD patients compared to that of controls^50^. Pro-inflammatory cytokines, including TNF-α, were also overexpressed in colonic biopsies from PD patients^18^. Thus, these pathological changes induced by *P. mirabilis* may represent the condition in the colon of PD patients.

Then, we assessed whether *P. mirabilis* induced overexpression of α-synuclein, a pathological hallmark protein in PD, in the brain and in the colon. In an *in vitro* experiment, *P. mirabilis* treatment increased the mRNA levels of α-synuclein significantly in SH-SY5Y cells. Although we could not clarify whether the effect was induced by *P. mirabilis* itself or LPS generated from the microbiota, this finding indicates that *P. mirabilis* may be involved in the production of α-synuclein in dopaminergic neurons. We also demonstrated the increase of α-synuclein aggregates both in the colon and in the SN of mice after *P. mirabilis* treatment. Pan-Montojo and his colleagues reported that α-synuclein first appeared in enteric neurons, moved into the dorsal motor nucleus of the vagus, and was eventually detected in the SN of rotenone-induced PD-like mice^37^. According to Braak theory, α-synuclein migrates from the gastrointestinal tract to the brain and overexpression of α-synuclein starts in the intestine^51,52^. Our results in VGX mice showed the consistent with the previous results by Holmqvistet and his colleagues that aggregated α-synuclein that was injected directly into the intestine migrated from the intestinal wall to the brain via the vagal nerve^16^. It has been also reported that inflammatory factors such as LPS and pro-inflammatory cytokines may trigger aggregation and accumulation of α-synuclein in enteric nerves^19,53^. Based on these reports, we expect that the increased α-synuclein aggregates by *P. mirabilis* treatment in the distal colon may move to the SN where it triggered dopaminergic neuronal damage and neuroinflammation. Additionally, increased pro-inflammatory cytokine TNF-α due to LPS insult in the colon may move directly via the blood to the SN and trigger α-synuclein aggregation and dopaminergic neuronal damage. Several studies suggest that other virulence factors of *P. mirabilis* except LPS may involve in intestinal inflammation or increased intestinal permeability. For example, flagella of *P. mirabilis* allows invasion into colonic epithelial barrier, thereby induces the increase of permeability and *P. mirabilis*-generated mannose-resistant Proteus-like fimbriae, which is closely related to biofilm formation as an adhesion factor, exhibits cytotoxicity on eukaryotic epithelial cells^54,55^. Meanwhile, it is not possible to exclude the possibility that other gut microbiomes except *P. mirabilis* could be involved in PD pathology in *P. mirabilis*-treated mice. For example, Chen *et al*. indicated the causal relationship between *E. coli*-producing curli amyloid and α-synuclein pathology^24^. Further researches on the actions of other bacteria after *P. mirabilis* administration in mice are needed.

In conclusion, we demonstrated that *P. mirabilis*, which showed increased bacterial colonies in the feces of three PD mouse models, is pathogenic in these animal models. It induced PD-related pathological changes, including dopaminergic neuronal death, neuroinflammation, and α-synuclein aggregation in the brain. We suggest that these pathological changes may be due to LPS as a *P. mirabilis*-generated virulence factor in the colon. Here, our findings are meaningful in that they provide the experimental evidence that a specific gut bacterium, which is commonly increased in PD mice, may cause the phenotype and pathogenesis of PD. It is required that further exploration to clarify the relevance of *P. mirabilis* for PD pathology under the conditions with germ-free or *P. mirabilis*-specific antibiotics treatment.

## Methods

### Animals

The mice used in the study were purchased from Daehan Biolink (Eumseong, Korea) and included the following: male C57BL/6 mice; 12-week-old (MPTP/p-induced PD mice) and 7-week-old mice (MPTP-induced PD mice, mice administrated with MPTP + *P. mirabilis*, mice with intra-rectal injection of LPS_P. mirabilis_, and mice with administrated *P. mirabilis* alone), male ICR 6-week-old mice (6-OHDA-induced PD mice). After the adaptation periods for 7 days, mice were housed in separate cages per each group (n = 4 per cage) at an ambient temperature of 23 ± 1 °C and relative humidity 60 ± 10% under a 12 h light/dark cycle and were allowed free access to water and food. All animal studies were performed in accordance with the “Principles of Laboratory Animal Care” (NIH publication number 80–23, revised 1996) and approved by the “Animal Care and Use Guidelines” of Kyung Hee University, Seoul, Korea (the approval number: KHUASP(SE)-16-132 and KHUASP(SE)-17-004). No research involved human participants in this study.

### MPTP/p-induced PD model

The MPTP/p-induced PD model was made according to previously described methods^56^. Detailed methods are described in Supplementary Methods.

### MPTP-induced PD model

The MPTP-induced PD model was made according to our previously reported methods^57^. Briefly, mice were injected with MPTP hydrochloride (30 mg/kg/day in saline, *i.p*. for 5 days). Vehicles of equal volume (0.25 ml) were given to the normal group. Feces were obtained at the 7^th^ day after the last MPTP injection.

### 6-OHDA-induced PD model

The 6-OHDA-induced PD model was made according to previously reported methods with some modification^58^. Briefly, mice were anesthetized, and two stereotaxic injections of 6-OHDA (16 μg/2 μl; 6-OHDA group), or an equivalent volume of 0.9% saline containing 0.2% ascorbic acid, were administered into the right ST (normal group), at a rate of 1 μl/min (AP + 0.7, ML + 2.0, DV −3.4 mm from bregma and dura)^59^. Feces were obtained at the 21^st^ day after 6-OHDA stereotaxic injection.

### Isolation and culture of fecal microbiota

Two or three fresh stools per mouse (approximately 0.08–0.12 g, 0.04 g per individual pellet) was septically collected from MPTP/p, MPTP, 6-OHDA-induced mice, and each normal mice, respectively. Each stool was suspended in a diluted anaerobic broth (Nissui Pharmaceutical Co., Japan), inoculated in deoxycholate hydrogen sulphide lactose (DHL) for isolation of *Enterobacteriaceae* colonies (Eiken, Tokyo, Japan), and cultured anaerobically at 37 °C for 2 days. The grown colonies in the DHL agar plates were identified as *P. mirabilis* using 16 S rRNA gene sequencing.

### Isolation of LPS extracted from *P. mirabilis*

LPS_P. mirabilis_ was extracted using the hot phenol-water method as a previously reported method with several modifications^60^. Detailed methods are described in Supplementary Methods.

### Cell culture and *P. mirabilis* treatment *in vitro*

SH-SY5Y neuroblastoma cells were purchased from the Korean Cell Line Bank (Seoul, Korea). SH-SY5Y cells were cultured at 37 °C and 5% CO_2_ in Dulbeco’s Modified Eagle’s medium (Gibco, USA) supplemented with 10% heat-inactivated fetal bovine serum (PAN biotech GmbH Aidenbach, Germany). Detailed methods are described in Supplementary Methods.

### *P. mirabilis* administration *in vivo*

*P. mirabilis* was grown in tropic soy broth (200 ml) and harvested by the centrifuge at 10,000 × g for 10 min. Cell supernatants were then washed twice with phosphate buffered saline (PBS). Detailed methods are described in Supplementary Methods.

### Intra-rectal injection of LPS_P. mirabilis_ in mice

We performed intra-rectal administration of LPS_P. mirabilis_ according to the previously reported method^61^. Detailed methods are described in Supplementary Methods.

### Behavior test

We performed three motor behavior tests in this study. Detailed methods are described in Supplementary Methods.

### Biological sample preparation

For immunohistochemical studies, at 24 h after behavioral tests, mice were perfused transcardially with 0.05 M PBS, and then fixed with cold 4% paraformaldehyde (PFA) in a 0.1 M phosphate buffer. Brains were removed and post-fixed in a 0.1 M phosphate buffer containing 4% PFA overnight at 4 °C and then immersed in a solution containing 30% sucrose in 0.05 M PBS for cryoprotection. Serial 30 µm-thick coronal sections were cut on a freezing microtome (Leica, Germany) and stored in cryoprotectant (25% ethylene glycol, 25% glycerol, and 0.05 M phosphate buffer) at 4 °C until use. For western blot analysis, the mice were decapitated and the brains or distal colons were isolated and stored at −80 °C until use. We selected the distal region of the colon due to the large amount of intestinal bacteria in this region^62^.

### Determination of LPS in feces and serum

The LPS content of the feces, serum, and brain cortex was determined using diazo-coupled LAL assays. Briefly, the feces (approximately 0.08–0.12 g) collected from the mice were placed in 50 ml of PBS in a pyrogen-free tube, sonicated for 1 h on ice, and sterilized by filtration through a 0.45-μm filter followed by re-filtration through a 0.22-μm filter. The serum (5 μl) was diluted 1:10 in pyrogen-free water and heated at 70 °C for 10 min. The LPS levels in the feces and serum were assayed using the LAL Assay Kit (Cape Cod Inc., USA), according to the manufacturer’s protocol.

### Immunohistochemistry

For the immunohistochemical study, the brain sections were selected according to mouse brain atlas (SN; from −2.92 to −3.52 mm, ST; from 1.18 to 0.38 mm, HP and primary sensory cortex (PSC); from −1.94 to −2.30 mm following coordinates from the bregma)^63^. Detailed methods are described in Supplementary Methods. The images were photographed using an optical bright-field or fluorescence microscope (BX51, Olympus, Japan).The number of tyrosine hydroxylase (TH) and ionized calcium-binding adapter molecule 1 (Iba1) positive cells in the SN, ST, HP, and PSC was quantified according to stereological counting^64^ and they were analyzed with Image J software.

### Dot blotting

Dot blotting was performed according to previously described methods^65^. For dot blot quantification of α-synuclein filament, 8 μg samples of the distal colon were spotted on an Immobilon-P transfer membrane and subsequently blocked for 30 min with 5% skim milk in TBST. After rinsing with TBST, the membrane was incubated with a primary antibody for 1 h at room temperature and then treated with an HRP secondary antibody for 30 min. Immuno-reactive bands and their quantification were equally measured as the methods of western blotting.

### Quantitative real time-polymerase chain reaction

Total RNA was extracted from SH-SY5Y cells, using a total RNA extraction kit (Qiagen, Germany). Synthesis of cDNA for detection of α-synuclein, and glyceraldehyde 3-phosphate dehydrogenase (GAPDH) was performed with 2 μg of total RNA, oligo(dT) primers (α-synuclein, forward: 5′-AGGCAGCTGGAAAGACAAAA-3 and the reverse: 5′-CAGCTCCCTCCACTGTCTTC-3′; and GAPDH, forward: 5′-TGCAGTGGCAAAGTGGAGAT-3′ and the reverse: 5′-TTTGCCGTGAGTGGAGTCATA-3′), and a reverse transcriptase in a total volume of 40 μl, as described previously. PCRs were performed in a total volume of 50 μl, comprising 4 μl of cDNA product and 25 μl of Premix EX Taq (TaKaRa Bio Inc., Japan), using a TaKaRa thermal cycler and SYBR premix agents, per the instructions provided by TaKaRa. Thermal cycling conditions were as follows: activation of DNA polymerase at 95 °C for 5 min, followed by 40 cycles of amplification at 95 °C for 10 sec and at 60 °C for 30 sec. Gene expression was normalized with respect to GAPDH.

### Statistical Analysis

All statistical parameters were calculated using Graphpad Prism 5.0 software (GraphPad Software, Inc., USA) or SPSS 11 software (SPSS Inc., USA). Values were expressed as the mean ± standard error of the mean (SEM). All results were evaluated by the one-way ANOVA analysis (Tukey’s *post hoc* test) or unpaired t-test. Differences with a *p*-value less than 0.05 were considered statistically significant. Data that is out of range of mean ± 2-fold standard deviation was excluded. All experiments were repeated for reproducibility.

### Data availability

The datasets generated during and/or analyzed during the current study are available from the corresponding author on reasonable request.

## Electronic supplementary material

Supplementary information
