# Supplementary material for: Oral administration of Proteus mirabilis damages dopaminergic neurons and motor functions in mice
Source: Sci Rep. 2018 Jan 19;8:1275. doi: 10.1038/s41598-018-19646-x (PMC5775305; doi:10.1038/s41598-018-19646-x)

Supplementary information

Oral administration of *Proteus mirabilis* damages dopaminergic neurons and motor functions in mice

Jin Gyu Choi[1], Namkwon Kim[1], In Gyoung Ju[1], Hyeyoon Eo[1], Su-Min Lim[1], Se-Eun Jang[1], Dong-Hyun Kim[1,*], Myung Sook Oh[1,2,*]

[1]Department of Life and Nanopharmaceutical Sciences, Graduate School, Kyung Hee University, 26, Kyungheedae-ro, Dongdaemun-gu, Seoul, 02447, Republic of Korea

[2]Department of Oriental Pharmaceutical Science, College of Pharmacy and Kyung Hee East-West Pharmaceutical Research Institute, Kyung Hee University, 26, Kyungheedae-ro, Dongdaemun-gu, Seoul, 02447, Republic of Korea

[*]To whom correspondence should be addressed.

E-mail address: msohok@khu.ac.kr (Oh MS), dhkim@khu.ac.kr (Kim DH)

Supplementary Methods

MPTP/p-induced PD model. Mice were injected with MPTP hydrochloride (25 mg/kg/day in saline, *i.p.*) along with probenecid (100 mg/kg/day in 5 % $NaHCO_3$, *i.p.*) to induce parkinsonian symptoms. The normal group were injected with equal volume of sterilized saline. Probenecid was administered 30 min prior to MPTP administration as it decreases the clearance of MPTP and intensifies its neurotoxicity. These mice received a total of 10 injections of MPTP in combination with probenecid. The 10 injections were given at an interval of 3.5 days between consecutive doses for a 5-week schedule. Feces were obtained after the 10th injections of MPTP/p (Fig. 1B).

Isolation of LPS extracted from *P. mirabilis*. In brief, broth media of *P. mirabilis* (100 ml) was centrifuged at 10,000 ×g for 10 min. The pellets were washed twice in PBS (pH 7.2) and sonicated for 10 min on ice. In order to remove protein and nucleic acids, treatment with proteinase K, DNase and RNase was performed prior to the extraction process. For this purpose, proteinase K (100 µg/ml) (Sigma, USA) was treated to bacterial cells, and the cells were kept at 65 °C for 1 h. The mixture was then treated with RNase (40 µg/ml) (Sigma, USA) and DNase (20 µg/ml) (Sigma, USA) in the presence of 20% $MgSO_4$ (1 µl/ml) and chloroform (4 µl/ml), and incubation was performed at 37 °C for 14 h. Next, an equal volume of hot phenol was added to the mixtures followed by shaking the incubation at 65-70 °C for 15 min. Suspensions were then cooled, transferred to 1.5 ml micro-tubes and centrifuged at 10,000 ×g for 15 min. Cell supernatants were transferred to 15 ml conical tubes, and phenol phases were re-extracted by distilled water. $NaCH_3CO_2$ at 0.5 M final concentration and 10 volumes of 95% EtOH were added to the mixtures, which were stored at -20 °C overnight in order to precipitate $LPS_{P. mirabilis}$. Tubes were then centrifuged at 2,000 ×g 4 °C for 10 min, and the pellets were re-suspended in 1 ml distilled water. The washing process was repeatedly performed until the residual phenol in the aqueous phases was totally eliminated.

Cell culture and *P. mirabilis* treatment *in vitro*. The culture medium was changed every two days,

and the cells were passaged when they reached 80 to 90% confluency. To evaluate the effect of *P. mirabilis* on α-synuclein mRNA expression, the collected SH-SY5Y cells (1.5×10^6 cells/well) were seeded and treated with *P. mirabilis* ($\times 10^3$ and $\times 10^5$ colony-forming unit; CFU) or LPS (positive control; 10 and 100 ng/ml) for 24 h.

P. mirabilis* administration *in vivo. The collected *P. mirabilis* was orally administrated to mice for 5 days (2×10^8 CFU/0.2 ml PBS per mice). The mice were sacrificed at 8th and 16th day after last administration of *P. mirabilis*, respectively. In addition, to investigate the pharmacological influence of *P. mirabilis* on aggravation of MPTP neurotoxicity, the mice were injected with a half dose of MPTP hydrochloride (15 mg/kg/day in saline, *i.p.* for 5 days) together with *P. mirabilis* administration (*p.o.* for 5 days). The mice were sacrificed at 16th day after last administration of *P. mirabilis*. Normal group was orally treated with equal volume of PBS for 5 days.

Intra-rectal injection of $LPS_{P.\ mirabilis}$ in mice. In brief, 50 μl of $LPS_{P.\ mirabilis}$ (50 μg per mouse) was injected by rectal enema after anaesthetization using a disposable zonde which was advanced into the colon until the tip of the zonde was totally inserted. The vehicle-treated group was injected with equal volume of PBS. We ensured that there was no leakage after the procedure. The mice were fasted 24 h before $LPS_{P.\ mirabilis}$ injection and sacrificed at 16th day after $LPS_{P.\ mirabilis}$ injection.

Unilateral cervical vagotomy. Vagotomy was performed under anesthesia as described methods [1,2]. In brief, male 7-week-old mice were incised in the anterior neck skin to localize the right vagus nerve bundle. The vagus nerve was separated carefully from the carotid artery and cut, and the skin was closed with surgical suture. Sham-operated mice were exposed the vagal bundle, but the vagus nerve was not cut. The VGX or sham-operated mice were placed for 5 days as recovery period with free access to water and food. After the recovery period, the collected *P. mirabilis* was orally administrated

to mice for 5 days (2×10^8 CFU/0.2 ml PBS per mice). The mice were sacrificed at 16th day after last administration of *P. mirabilis*. Sham-operated or only VGX group was orally treated with equal volume of PBS for 5 days.

Pole test. We performed the pole test on the 16th day after *P. mirabilis* administration or the last injections of MPTP. The mice were held on the top of the pole (diameter 8 mm, height 55 cm, with a rough surface). The time needed for the mice to climb down and place all four feet on the floor was recorded with a 30 s cut-off limit. Each trial had a cut off limit of 30 s.

Open field test. The open field test was performed between 9 p.m. and 2 a.m. to avoid diurnal variation. The mice were placed in the testing chamber (40 × 25 × 18 cm) with white floors, followed by a 30-min recording period using a computerized automatic analysis system (Viewer; Biobserve, Bonn, Germany). The data collected by computer included the total distance traveled by tracking the center of the animal.

Rotarod test. We performed the rotarod test on the 16 days after last administration of *P. mirabilis* or MPTP. The rotarod unit consists of a rotating spindle (7.3 cm diameter) and five individual compartments. After two or three times of training (8-10 rpm rotation speed), the rotation speed was increased to 16 rpm in a test session. The time each mouse remained on the rotating bar was recorded over three trials per mouse with a maximum length of 3 min per trial. Data are presented as the mean time on the rotating bar over the three test trials.

Biological sample preparation. For immunohistochemical studies, at 24 h after behavioral tests, mice were perfused transcardially with 0.05 M PBS, and then fixed with cold 4% paraformaldehyde (PFA) in a 0.1 M phosphate buffer. Brains were removed and post-fixed in a 0.1 M phosphate buffer containing

4% PFA overnight at 4 °C and then immersed in a solution containing 30% sucrose in 0.05 M PBS for cryoprotection. Serial 30 µm-thick coronal sections were cut on a freezing microtome (Leica, Germany) and stored in cryoprotectant (25% ethylene glycol, 25% glycerol, and 0.05 M phosphate buffer) at 4 °C until use. For western blot analysis, the mice were decapitated and the brains or distal colons were isolated and stored at −80 °C until use.

Immunohistochemistry. The brain sections were briefly rinsed in PBS and treated with 1% hydrogen peroxide for 15 min. The sections were incubated with a rabbit anti-TH antibody (1:1000, AB152, Merck Millipore) for SN and ST tissues and a mouse anti-NeuN antibody (1:1000, MAB377, Merck Millipore) for hippocampal tissues overnight at 4 °C in the presence of 0.3% triton X-100. After rinsing in PBS, the sections were then incubated with biotinylated anti-rabbit, anti-mouse IgG (1:200, Vector Laboratories) for 1 h and with avidin-biotin complex mixture (1:100, PK-6100, Vector Laboratories) for 1 h at room temperature. Peroxidase activity was visualized by incubating sections with 3,3'-diaminobenzidine in 0.05 M tris–buffer. After several rinses with PBS, the sections were mounted on gelatin-coated slices, dehydrated and cover-slipped using a histomount medium. For measurement of the optical density of TH-positive area in the ST or NeuN-positive area in the hippocampal CA3 regions, the total region of interest was manually outlined and averaged optical densities were acquired in images with converted eight-bit indexed color. They were analyzed with Image J software (Bethesda, USA). To examine the activation of microglia in the ST, HP, and PSC, the brain sections were washed with PBS and incubated with a goat anti-Iba-1 antibody (1:1000, AB5076, Abcam) overnight at 4 °C in the presence of 0.3% triton X-100. After rinsing in PBS, the sections were incubated with chicken anti-goat Alexa 488 (1:500, A21467, Thermo Scientific) for 1 h and then 4',6-diamidino-2-phenylindole staining was performed for 20 min. To examine the activation of microglia (TH+Iba-1) or the aggregation of α-synuclein (TH+α-synuclein filament) in the SN, double immunofluorescence staining was performed. The SN sections for TH+Iba-1 double staining were incubated with a rabbit anti-TH (1:1000) for 6 h at room temperature. After washing with PBS, the sections were incubated with a goat anti-rabbit IgG Cy3 conjugate Alexa 594 (1:500, DI-1549, Vector Laboratories) and then incubated with a goat anti-Iba-1 antibody (1:1000)

overnight at 4 °C. After rinsing in PBS, the sections were incubated with chicken anti-goat Alexa 488 (1:500) for 1 h. The sections for TH+α-synuclein filament double immunostaining were incubated with a goat anti-TH (1:1000, ab101853, Abcam) for 6 h at room temperature. After washing with PBS, the sections were incubated with chicken anti-goat Alexa 488 (1:500) for 1 h and then incubated with a rabbit anti-α-synuclein filament antibody (1:1000, ab209538, Abcam) for 24 h at 4 °C. The sections were incubated with goat anti-rabbit IgG Cy3 conjugate Alexa 594 (1:500, DI-1549, Vector Laboratories) for 1 h. All immunofluorescent sections were mounted with anti-fade fluorescent medium (Wako chemical, Japan).

Western blotting. The protein samples were transferred onto Immobilon-P transfer membranes (Merck Milipore, MA, USA), and the membranes were blocked with 5% skim milk in tris-buffered saline-0.01% Tween 20 (TBST). Then, the membranes were incubated overnight at 4 °C with primary antibodies as follows: TLR4 (1:500, SC-293072, Santa Cruz Biotechnology), TNF-α (1:500, SC-1348, Santa Cruz Biotechnology), occludin (1:500, 33-1500, Thermo Scientific), and β-actin (1:4000, A300-491A, Bethyl Laboratories). After that, the membranes were washed four times for 10 min with TBST, and the blots were incubated with respective horseradish peroxidase-conjugated (HRP) secondary antibodies for 1 h at room temperature. Thereafter, the membranes were washed again four times for 10 min with TBST.

Dot blotting. For dot blot quantification of α-synuclein filament, 8 μg samples of the distal colon were spotted on a polyvinylidene difluoride membrane and subsequently blocked for 30 min with 5% skim milk in tris-buffered saline with tween 20 (TBST). After rinsing with TBST, the membrane was incubated with primary antibody (1:10000, ab209538, Abcam) for 1 h at room temperature and then treated with HRP secondary antibody for 30 min.

qRT-PCR. Total RNA was extracted from SH-SY5Y cells, using a total RNA extraction kit (Qiagen,

Germany). Synthesis of cDNA for detection of α-synuclein, and GAPDH was performed with 2 μg of total RNA, oligo(dT) primers (α-synuclein, forward: 5’-AGGCAGCTGGAAAGACAAAA-3 and the reverse: 5’-CAGCTCCCTCCACTGTCTTC-3’; and GAPDH, forward: 5’-TGCAGTGGCAAAGTGGAGAT-3’ and the reverse: 5’-TTTGCCGTGAGTGGAGTCATA-3’), and a reverse transcriptase in a total volume of 40 μl, as described previously. PCRs were performed in a total volume of 50 μl, comprising 4 μl of cDNA product and 25 μl of Premix EX Taq (TaKaRa Bio Inc., Japan), using a TaKaRa thermal cycler and SYBR premix agents, per the instructions provided by TaKaRa. Thermal cycling conditions were as follows: activation of DNA polymerase at 95 °C for 5 min, followed by 40 cycles of amplification at 95 °C for 10 s and at 60°C for 30 s.

Supplementary Figures

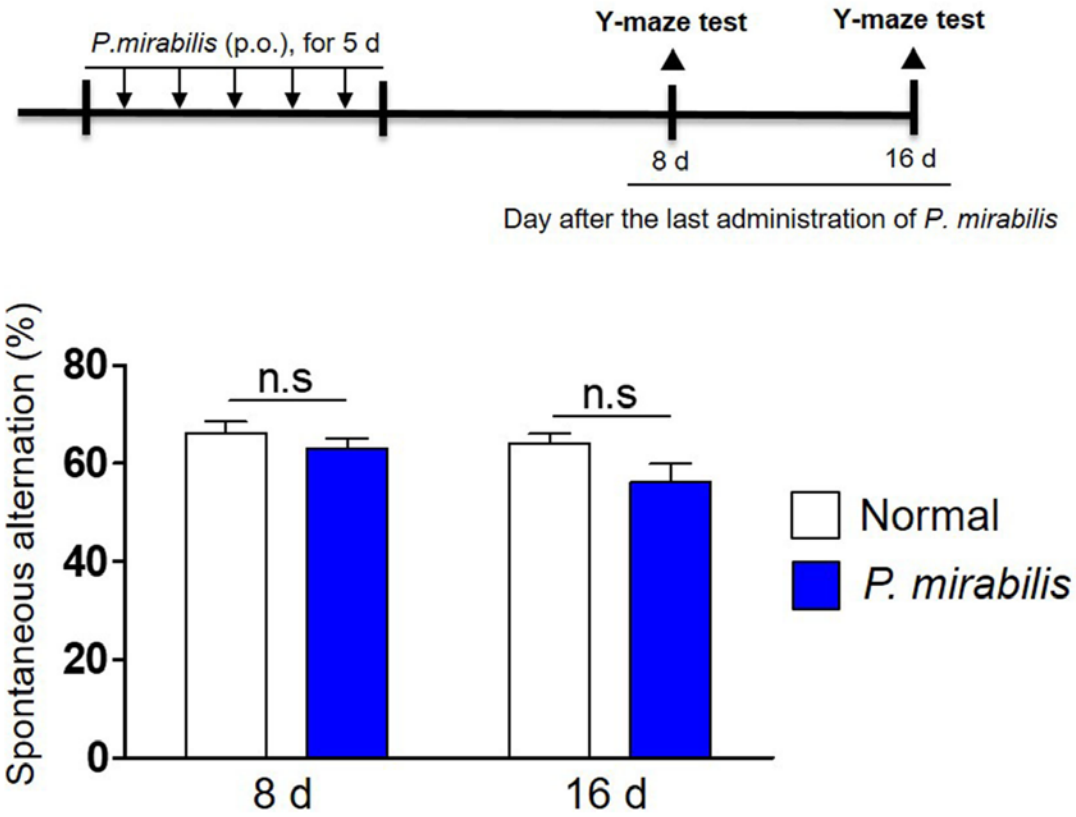

Figure S1. Memory function is unaffected by *P. mirabilis* treatment. Y-maze test was performed at 8th and 16th day after last administration of *P. mirabilis*. Values were expressed as means ± SEM (unpaired t-test; n=12). n.s; no significant.

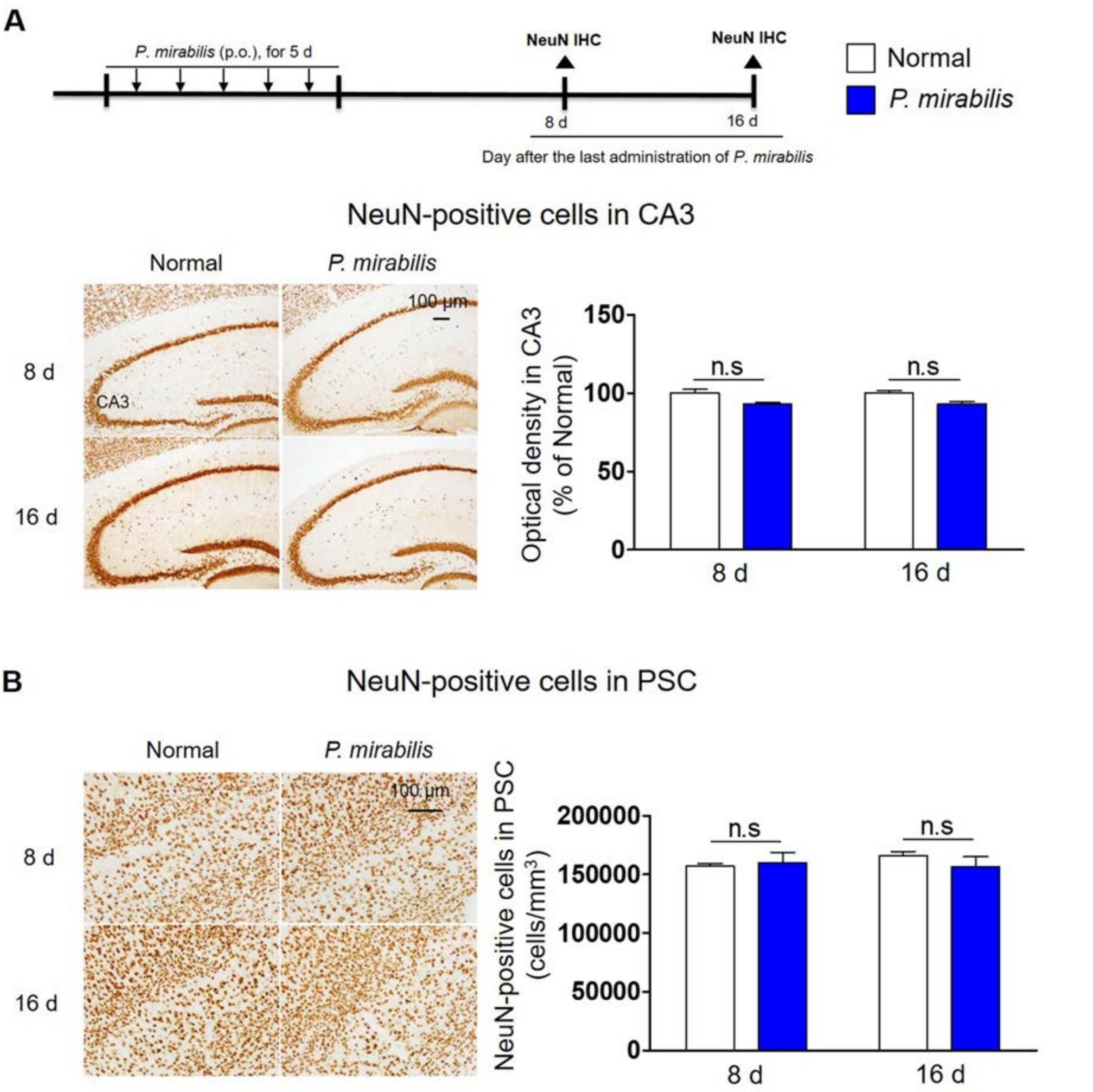

Figure S2. The hippocampal and cortical neurons are not damaged by *P. mirabilis* treatment. (A, B) The NeuN immunohistological analysis was performed at 8th and 16th day after last administration of *P. mirabilis* in hippocampal CA3 and PSC regions, respectively. Values were expressed as means ± SEM (unpaired t-test; n=8). n.s; no significant, NeuN; neuronal nuclei, IHC; immunohistochemistry, PSC; primary sensory cortex.

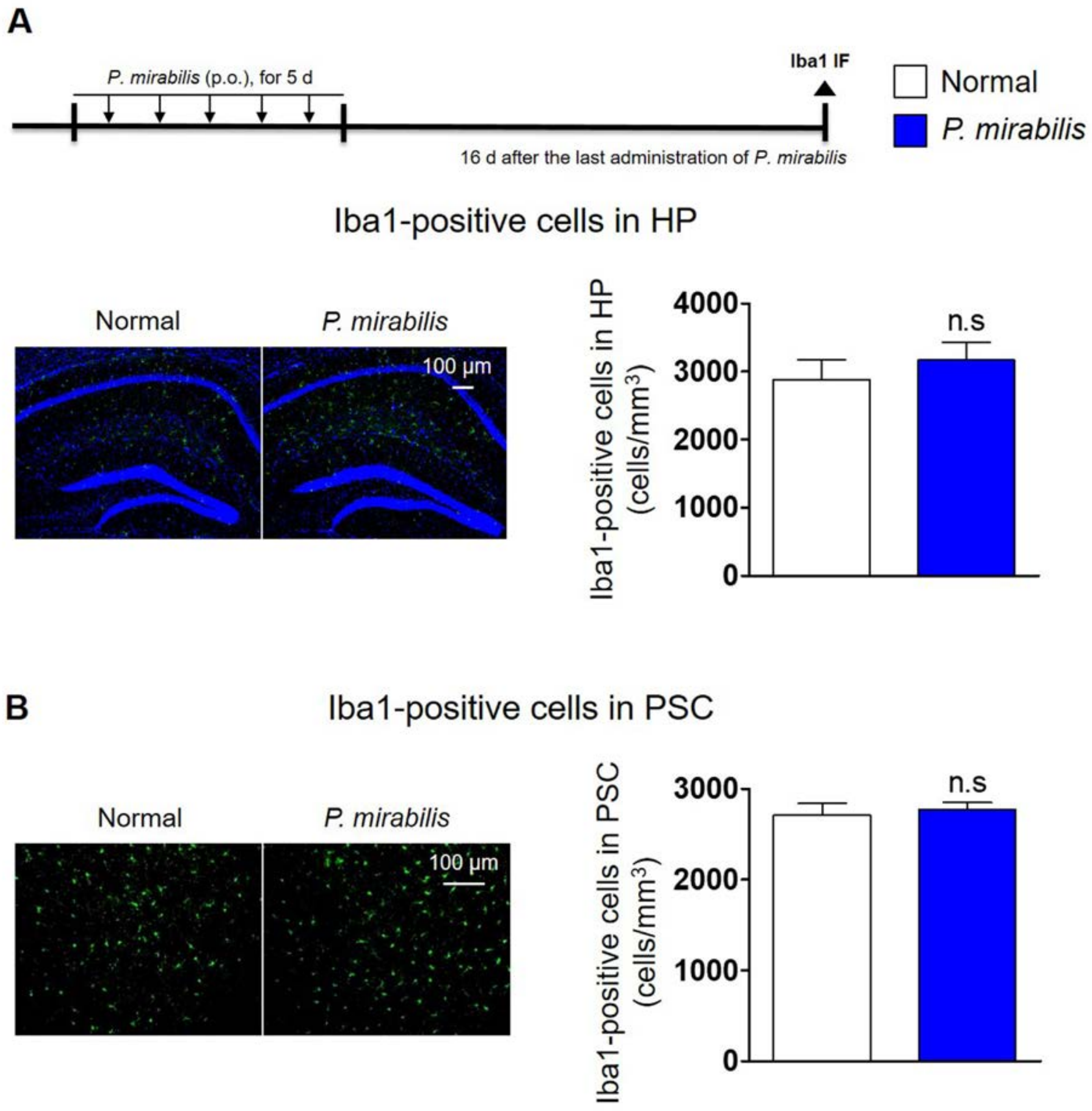

Figure S3. Neuroinflammation in the hippocampal and cortical regions of brain is not induced by *P. mirabilis* treatment. *(A, B)* The Iba1 immunohistological analysis was performed at 16th day after last administration of *P. mirabilis* in hippocampal and PSC regions, respectively. Values were expressed as means ± SEM (unpaired t-test; n=8). n.s; no significant, IF; immunofluorescence, HP; hippocampus, PSC; primary sensory cortex.

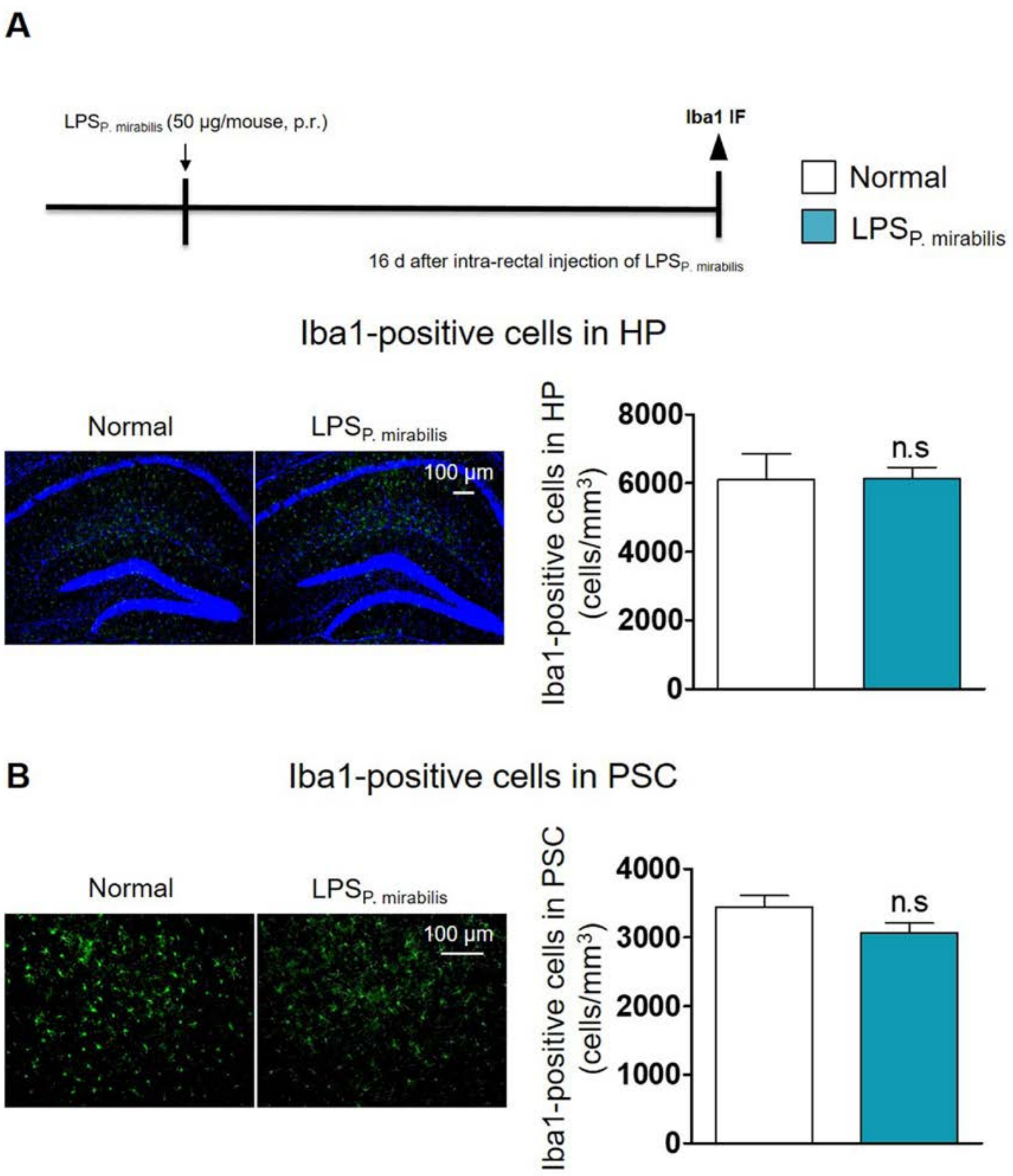

Figure S4. Intra-rectal injection of LPS derived from *P. mirabilis* does not induce inflammation in the hippocampal and cortical regions of brain. *(A, B)* The Iba1 immunohistological analysis was performed at 16th day after last administration of *P. mirabilis* in hippocampal and PSC regions, respectively. Values were expressed as means ± SEM (unpaired t-test; n=8). n.s; no significant, IF; immunofluorescence, HP; hippocampus, PSC; primary sensory cortex.

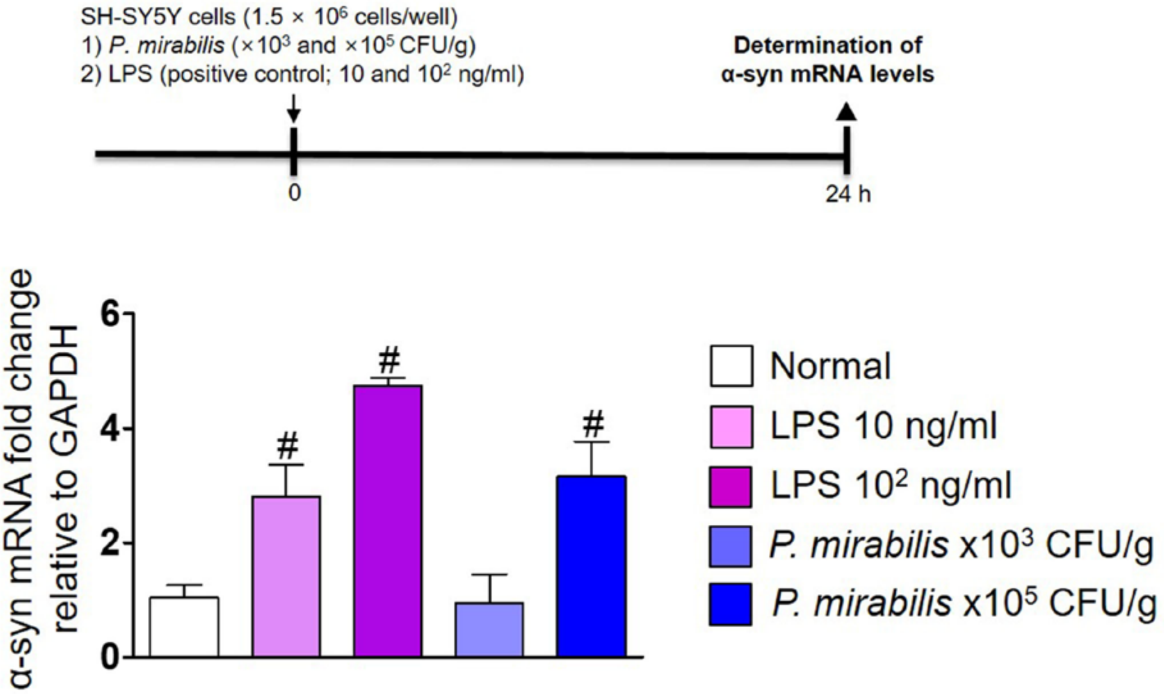

Figure S5. *P. mirabilis* treatment stimulates mRNA expression of α-synuclein in SH-SY5Y cells. The mRNA levels of α-synuclein were significantly increased in the *P. mirabilis*-treated group similar to those of LPS-treated group compared with normal group in SH-SY5Y cells. Values were expressed as means ± SEM. #$p<0.05$ vs. normal group (unpaired t-test; n=3). CFU; colony-forming unit, α-syn; α-synuclein.

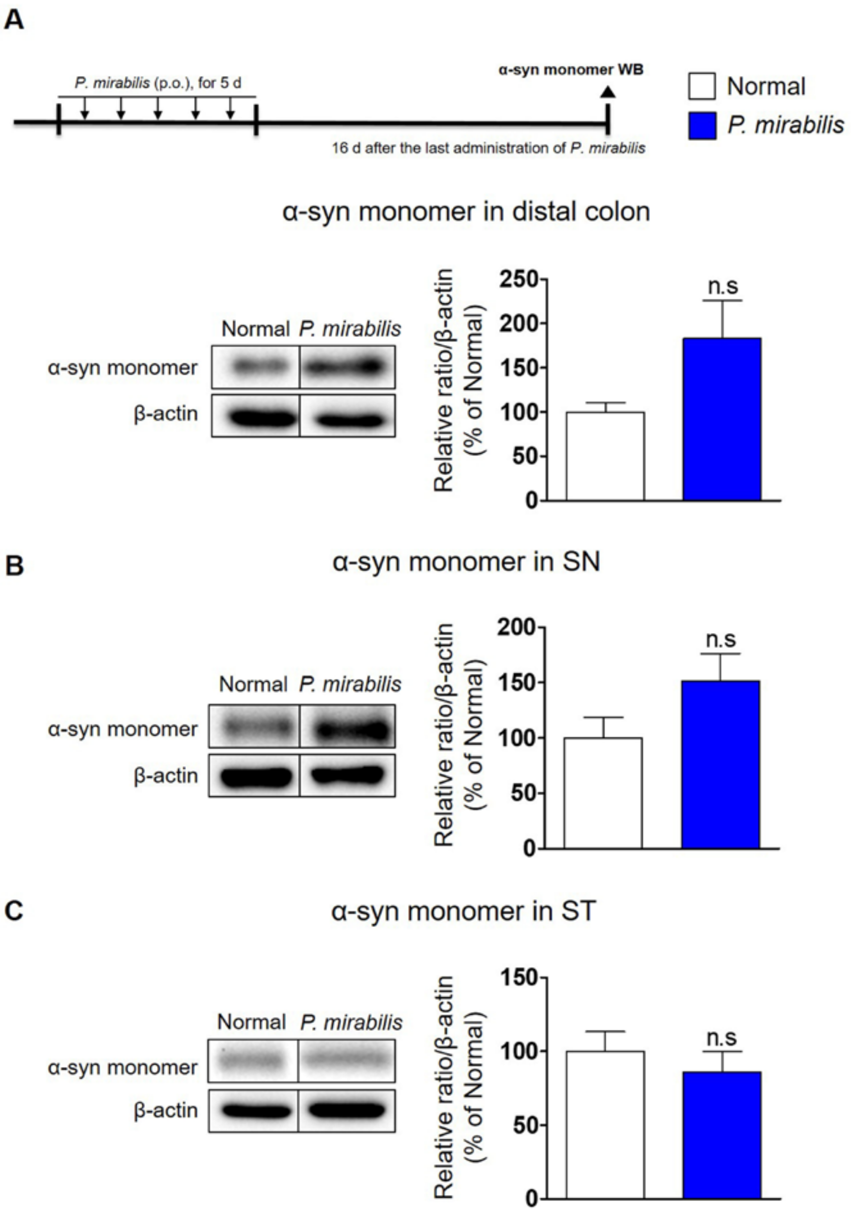

Figure S6. *P. mirabilis* treatment has few effects on the expression of α-synuclein monomer in SN and ST of brain. The blots were processed in parallel using the samples derive from the same experiment. *(A)* The protein levels of α-synuclein monomer were measured at 16th day after last administration of *P. mirabilis* in distal colon. *(B, C)* Those were also measured in SN and ST regions of brain at the same time point, respectively. Values were expressed as means ± SEM (unpaired t-test; n=4). n.s; no significant, WB; western blotting, α-syn; α-synuclein.

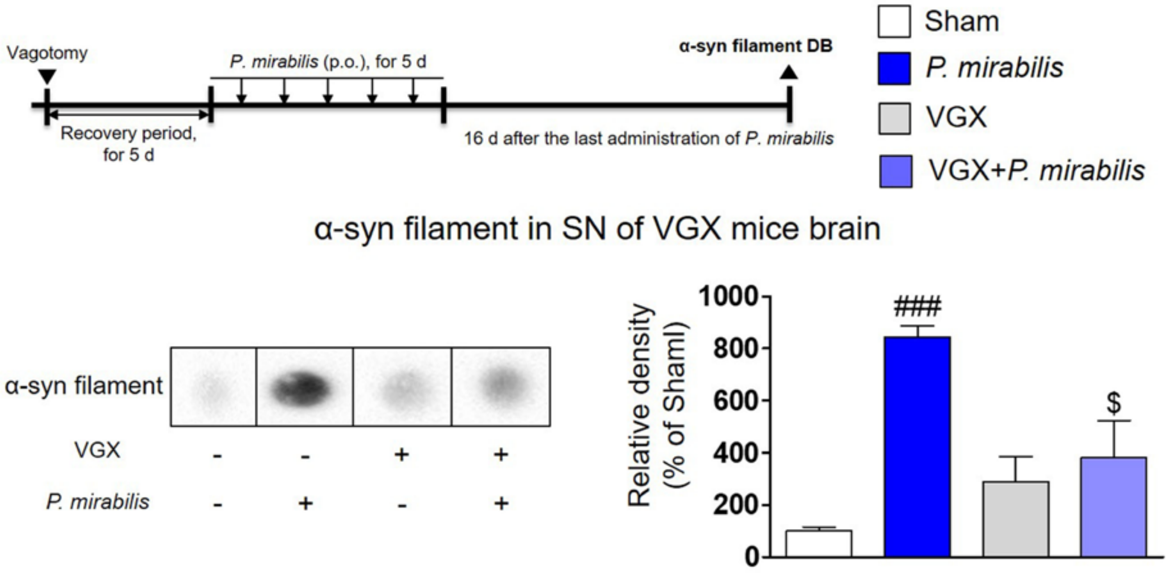

Figure S7. *P. mirabilis* treatment does not induce aggregation of α-synuclein in SN of vagotomized mice brain. The blots were processed in parallel using the samples derive from the same experiment. The protein levels of α-synuclein filament was measured in the SN of sham-operated or VGX mice brain. Values were expressed as means ± SEM. ###$p<0.001$ vs. sham-operated group; \$$p<0.05$ vs. *P. mirabilis* only treated group (unpaired t-test; n=3). DB; dot blotting, α-syn; α-synuclein.

References

1 Borovikova, L. V. *et al.* Role of vagus nerve signaling in CNI-1493-mediated suppression of acute inflammation. *Auton Neurosci.* **85**, 141-147 (2000).

2 Calleja-Castillo, J. M. *et al.* Chronic deep brain stimulation of the hypothalamic nucleus in wistar rats alters circulatory levels of corticosterone and proinflammatory cytokines. *Clin Dev Immunol.* **2013**, 698634, doi:10.1155/2013/698634 (2013).

The raw image of blotting

-The blotting band surrounded by a red-colored box was used as the representative image.

1. The image of blotting band in Fig. 6

<Occludin>

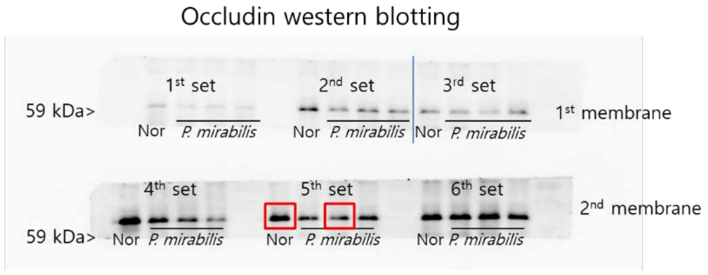

<TNF-α>

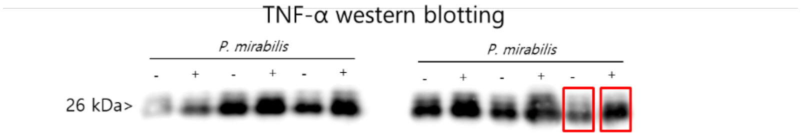

<TLR4>

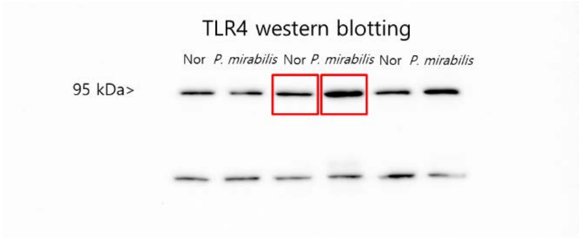

<β-actin>

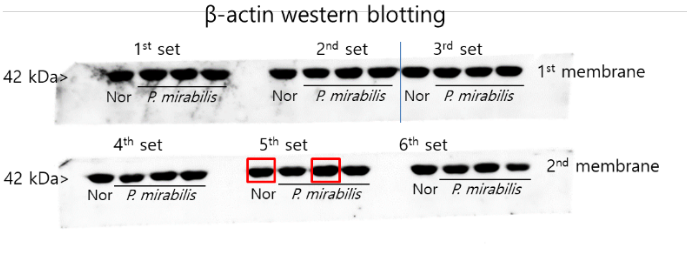

2. The image of blotting band in Fig. 8

<α-synuclein filament>

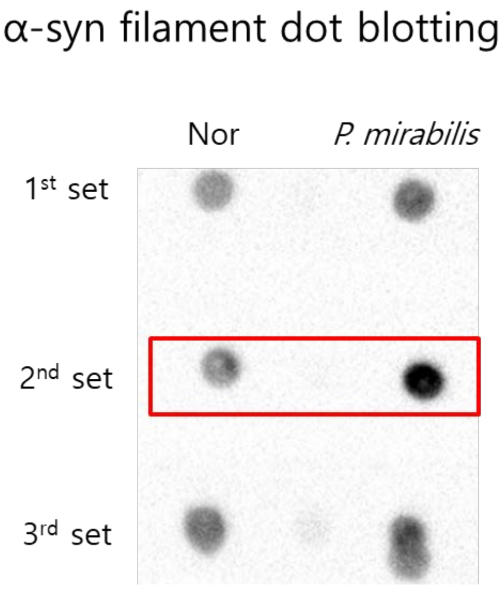

3. The image of blotting band in Fig. S6

<α-synuclein monomer and β-actin in distal colon>

α-syn monomer western blotting (Distal colon)

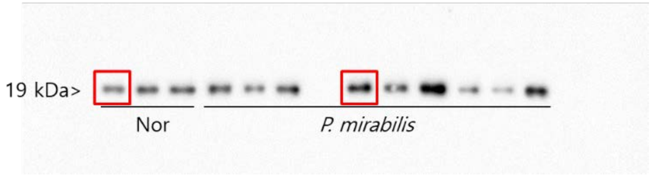

β-actin western blotting (Distal colon)

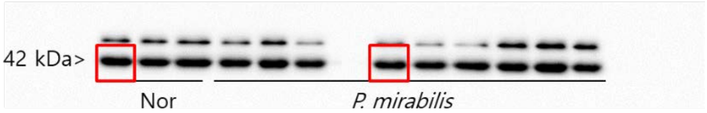

<α-synuclein monomer and β-actin in SN>

α-syn monomer western blotting (SN)

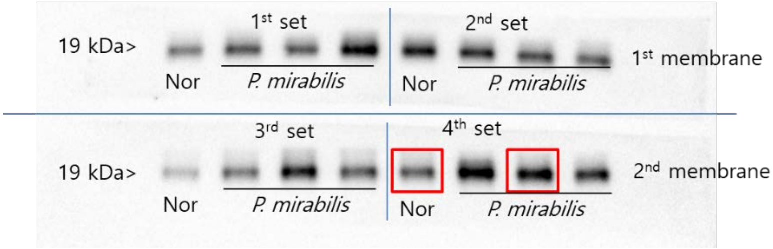

β-actin western blotting (SN)

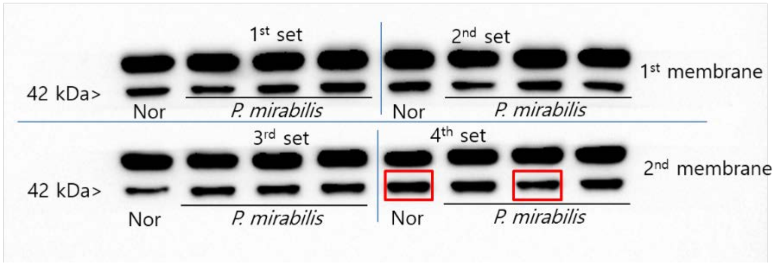

<α-synuclein monomer and β-actin in ST>

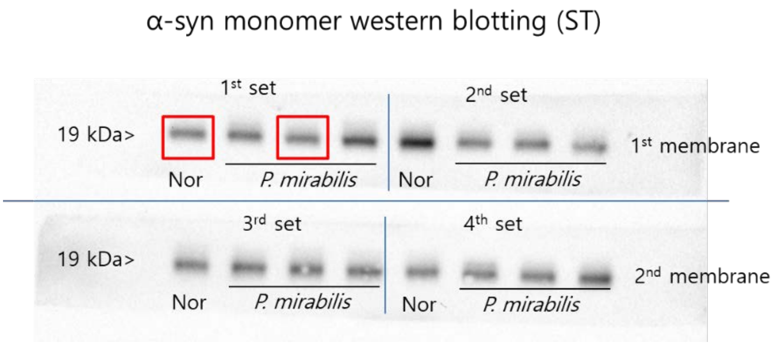

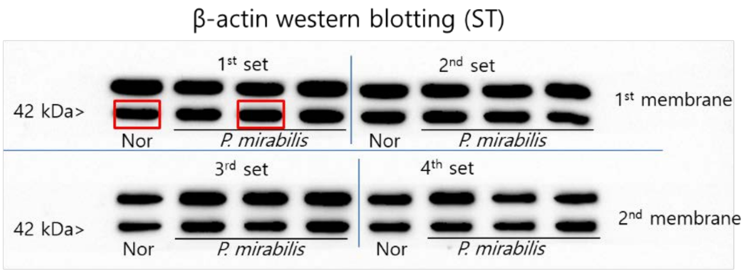

Supplement: Supplementary file 1 — Supplementary information [file 41598_2018_19646_MOESM1_ESM.pdf]
